# Artificial Intelligence Diagnosis of Obstructive Sleep Apnea Using Overnight Pulse Oximetry: A Systematic Review and Bayesian Meta-Analysis

**DOI:** 10.2196/80349

**Published:** 2026-07-08

**Authors:** Kvan Jie Ming Yam, Claire Yi Jia Lim, Esther Yanxin Gao, Jin Hean Koh, Nicole Kye Wen Tan, Adele Chin Wei Ng, Zhou Hao Leong, Chu Qin Phua, Thun How Ong, Leong Chai Leow, Guang-Bin Huang, Benjamin Kye Jyn Tan, Song Tar Toh

**Affiliations:** 1 Lee Kong Chian School of Medicine, Nanyang Technological University, Singapore, Singapore; 2 SingHealth Duke-NUS Academic Medical Centre, Singapore, Singapore; 3 School of Medicine, Faculty of Medical and Health Sciences, University of Auckland, Auckland, New Zealand; 4 Yong Loo Lin School of Medicine, National University of Singapore, Singapore, Singapore; 5 Surgery Academic Clinical Program, SingHealth, Singapore, Singapore; 6 Department of Otorhinolaryngology–Head & Neck Surgery, Singapore General Hospital, Block 3 Outram Rd, Basement 1 Singapore General Hospital, Singapore, 169608, Singapore, +65 6222 3322; 7 Duke-NUS Sleep Centre, SingHealth, Singapore, Singapore; 8 Department of Otorhinolaryngology–Head & Neck Surgery, Sengkang General Hospital, Singapore, Singapore; 9 Department of Respiratory and Critical Care Medicine, Singapore General Hospital, Singapore, Singapore; 10 School of Automation, Southeast University, Nanjing, China; 11 Key Laboratory of Measurement and Control of Complex Systems of Engineering, Ministry of Education, Nanjing, China; 12 Mind PointEye, Singapore, Singapore

**Keywords:** machine learning, neural networks, deep learning, sleep disordered breathing, sleep apnoeas, diagnostic test accuracy

## Abstract

**Background:**

Obstructive sleep apnea (OSA) affects 38% of the population, yet over 90% of cases remain undiagnosed. The gold standard for diagnosis, polysomnography, requires specialized equipment and trained personnel, making it inaccessible in primary care and acute settings. With artificial intelligence (AI) advancements, oximetry-based AI models have emerged as potential alternatives for OSA diagnosis.

**Objective:**

This meta-analysis aims to evaluate the diagnostic accuracy of AI models trained on pulse oximetry readings in diagnosing OSA.

**Methods:**

A systematic search was conducted across Medline/PubMed, Embase, Scopus, Web of Science, and IEEE Xplore databases from inception to January 3, 2026. Studies that evaluated the diagnostic accuracy of AI models trained on oxygen saturation recordings, compared to the apnea-hypopnea index (AHI) as the reference standard, were included and screened by 2 blinded independent reviewers. Models were evaluated using Bayesian bivariate meta-analysis and meta-regression. Publication bias was examined using a selection model approach, while risk of bias and evidence quality were assessed with Quality Assessment of Diagnostic Accuracy Studies-2 (QUADAS-2) and Grading of Recommendations Assessment, Development, and Evaluation (GRADE).

**Results:**

From 13,986 screened articles, 25 studies met the inclusion criteria, encompassing 23,171 participants with a mean age of 40 (SD 10.6) to 63 (SD 13.3) years and a BMI of 25 to 37 kg/m^2^. AI-oximetry models demonstrated a pooled sensitivity of 91.1% (95% credible interval [CrI] 89.7%‐92.4%) and specificity of 88.4% (95% CrI 85.3%‐90.8%). Neural network classifiers achieved the highest sensitivity (92.7%) and specificity (91.3%). Deep learning feature extraction models were significantly higher in sensitivity (by 3.7%; 95% CrI 0.9%-6.9%) than domain expert-based approaches. Sensitivity decreased slightly with higher AHI cutoffs, while specificity increased by 16.6% from an AHI cutoff of ≥5 to ≥30. Sensitivity analyses showed that even with up to 40% probability of an unpublished study, changes in accuracy were modest (area under the curve: 0.902 to 0.877). QUADAS-2 and GRADE assessments found low-moderate risk of bias with high overall quality of evidence.

**Conclusions:**

AI-oximetry models showed high diagnostic accuracy for OSA across models and AHI cutoffs, performing better than or comparably to traditional overnight oximetry and home sleep apnea tests. This review provides the first pooled quantitative synthesis of AI models trained solely on oximetry data, with additional evaluations of publication bias and methodological limitations. Prior reviews were largely narrative or used alternative AI inputs other than oximetry. This study advances the field by offering a clearer and more reliable evidence base on pooled AI oximetry performance. These findings support the potential of oximetry-based AI as a convenient and scalable tool for OSA screening and diagnosis, with potential real-world applications in both primary care and inpatient settings for early identification of high-risk patients. Prospective external validation in diverse populations and low-prevalence settings is still needed before widespread real-world use.

## Introduction

### Rationale

Obstructive sleep apnea (OSA) is a highly prevalent and yet underdiagnosed condition, with an estimated 38% prevalence in the general population, yet over 90% of patients remain undiagnosed [1]. During sleep, these patients experience recurrent upper airway obstruction, which results in intermittent oxygen desaturation and sleep disruption, which increase their risk of developing devastating health complications like heart disease, stroke, chronic kidney disease, cognitive decline, depression, and cancers [2-6]. The economic ramifications of OSA extend beyond their health sequelae but also include OSA-related fatigue causing lost productivity, work-related accidents, and motor vehicle accidents [7].

A major contributor to the persistent diagnostic gap is the reliance on overnight polysomnography, the gold-standard test for OSA. Polysomnography is resource-intensive, requiring an inpatient night stay with complex equipment and skilled technicians [8]. This test is thus limited in availability, particularly in the primary care setting or in low-middle-income countries, where the majority of the world’s population lives [9]. Simple and convenient screening tools such as the STOP-BANG (Snoring, Tiredness, Observed apnoea, Pressure [hypertension], BMI>35 kg/m^2^, Age>50, Neck circumference>40 cm, and Gender [male]) questionnaire that have been widely used could help to address this need for OSA risk stratification in the primary care setting. However, while it has a high sensitivity of over 90%, it has a low specificity of 28%, which results in many false positives (FP) [10]. As such, many patients would be expected to screen positive with STOP-BANG, and with polysomnography as the only diagnostic tool, this would still result in a high number of undiagnosed cases. As health care systems globally are strained, this results in a long wait time for polysomnography appointments and time to treatment initiation. Such delays can negatively impact patient outcomes. For example, a center in Calgary, Canada, reported a mean time to treatment of 123 days and found that longer wait times were associated with decreased adherence to treatment and smaller improvements in daytime sleepiness assessed using the Epworth Sleepiness Scale score [11].

As the artificial intelligence (AI) sector advances rapidly, pulse oximetry readings have become a viable input for AI-guided diagnosis of OSA [12-14]. Overnight peripheral pulse oximetry for oxygen saturation (SpO_2_) is a simple, noninvasive, and widely available tool already used commonly in select clinical settings to diagnose OSA when a polysomnogram may not be feasible or acceptable, such as in pediatrics. It is physiologically relevant to OSA diagnosis because apneic and hypopneic events cause characteristic episodic drops in arterial oxygen saturation [15]. Conventional quantitative indices derived from SpO_2_, such as the oxygen desaturation index (ODI), correlate strongly with the apnea-hypopnea index (AHI) measured by polysomnography, the gold standard for OSA diagnosis. While ODI-based diagnosis yields high sensitivity (>90%), its specificity remains modest between 40%‐60%, limiting its standalone diagnostic utility [16,17].

SpO_2_ measurement is integral to the formal definition of hypopnea, underscoring its diagnostic relevance [18]. Furthermore, the pattern and severity of nocturnal hypoxemia, as measured by SpO_2_, are associated with OSA-related morbidity, including neurocognitive impairment and cardiovascular risk [19]. However, oximetry alone cannot differentiate central versus obstructive events, nor can it identify sleep stages, constraining its standalone diagnostic value. An AI-driven analysis of SpO_2_ data—leveraging machine learning or deep learning techniques—may overcome these limitations by extracting complex features from desaturation patterns such as central tendency, morphology, frequency, and amplitude of desaturation to more accurately classify OSA severity [20]. This approach has the potential to provide scalable, low-cost, and automated diagnostic support that could ease pressure on sleep laboratories, facilitate earlier identification of at-risk individuals, and improve access to care in underserved regions.

Despite the growing number of studies on AI in diagnosing OSA, evidence remains fragmented. Individual studies vary widely in model type, training methods, input features, oximeter brands and specifications, and diagnostic thresholds, and no comprehensive synthesis has evaluated the pooled diagnostic performance of AI models trained on SpO_2_ data. There remains a significant gap in the literature; a meta-analysis of the diagnostic capabilities of the models. Such a review is vital in assessing the accuracy, robustness, and clinical applicability of AI-driven oximetry for diagnosing OSA. To address the existing gaps, we conducted the first Bayesian meta-analysis evaluating the diagnostic accuracy of AI models trained on SpO_2_ recordings for OSA detection.

### Objectives

We aim to (1) quantitatively pool diagnostic performance metrics of AI models across studies and (2) use meta-regression to identify methodological and clinical factors associated with higher diagnostic accuracy. We hypothesize that AI models may be able to support risk stratification, thereby reducing the demand for polysomnography, while acknowledging that with the current capabilities of AI, it is unlikely to replace polysomnography as the definitive diagnostic modality.

## Methods

### Information Sources

The prespecified protocol for this review was registered on PROSPERO (International Prospective Register of Systematic Reviews; CRD42025648556). With reference to the PRISMA (Preferred Reporting Items for Systematic Review and Meta-Analyses) 2020 expanded guidelines and PRISMA-S (Preferred Reporting Items for Systematic Reviews and Meta-Analyses Literature Search Extension) checklist (Checklists1  2), a search was conducted on Ovid Medline/PubMed, Elsevier Embase, Elsevier Scopus, Clarivate Web of Science, and IEEE Xplore databases for studies published from inception till 3 January 2026 [21-23]. Multidatabase searching on a single platform was not performed. The PRISMA 2020 Expanded checklist is included in Checklist 3.

### Search Strategy

The search strategy was adapted from a previous review on machine listening for OSA diagnosis by the same corresponding author [24], which used a combination of the following search terms: (“sleep apnea” OR “sleep apnoea” OR “nocturnal hypoxia” OR “nocturnal hypoxaemia” OR “nocturnal hypoxemia” OR “sleep disordered breathing”) AND (“artificial intelligence” OR “machine learning” OR “deep learning” OR “logistic regression” OR “support vector machine” OR “neural network” OR “classification tree” OR “regression tree” OR “probability tree” OR “nearest neighbor” OR “nearest neighbor” OR “fuzzy logic” OR “naive bayes” OR “genetic algorithm” OR “multilayer perceptron” OR “random forest” OR “lasso regression” OR “kernel regression” OR “elastic net” OR “generative model” OR “generative adversarial network” OR “large language model”) AND (diagnosis OR diagnose OR detect OR detection OR identify OR identification OR severity OR classify OR classification). The full search strategies for each database are available in Multimedia Appendix 1, including the description of any limits applied. Due to the extensive search strategy and large number of search results, no additional hand-searching through study registries, online browsing, citation searching, author contacts, or any other methods was performed. No search filters and no search peer review process were used.

### Managing Records

A total of 13,986 records were retrieved from the database search (PubMed: 3912; Embase: 1487; Web of Science: 3424; Scopus: 2780; IEEE Xplore: 2383). Software used for deduplication includes EndNote, Rayyan (Rayyan Systems Inc), and TERA the deduplicator [25-27]. After deduplication, 5551 duplicates were removed, leaving 8435 records for title and abstract screening.

### Selection Process

Records were uploaded onto Rayyan [27], an online systematic review platform that enables authors to manually screen abstracts in a blinded manner. Two blinded reviewers (KJMY and CYJL) independently screened the titles and abstracts, followed by full-text screening to check the eligibility for inclusion, with disputes being resolved by consensus from a third independent reviewer (JHK).

#### Eligibility Criteria

The inclusion criteria were as follows:

1. Population: adults aged at least 18 years
2. Intervention/exposure: diagnosis and classification of OSA using AI (traditional regression techniques, machine learning, etc) trained on SpO_2_ recordings from overnight polysomnography or home sleep apnea tests (HSATs).
3. Comparators: diagnosis and classification of OSA using the apnea-hypopnea index (AHI) from overnight polysomnography or HSATs.
4. Outcomes: Accuracy of AI in diagnosis and classification of OSA, assessed via a random split test set or k-fold cross-validation, and measured by sensitivity, specificity, positive predictive value, negative predictive value, and/or area under the curve (AUC).
5. Study type: observational studies (eg, cohort and cross-sectional).

The exclusion criteria were as follows:

1. Case reports, reviews, letters, conference abstracts, or other records not published as full-length articles in peer-reviewed journals.
2. Studies published in languages other than English that do not have an English translation.
3. Studies assessed as having a high risk of bias across 2 or more domains.
4. Studies that did not measure the diagnostic accuracy of AI in diagnosing AHI-defined OSA

#### Data Collection Process, Including Data Items

Data from included articles were extracted by 2 blinded, independent reviewers (KJMY and CYJL) in duplicate onto a structured form specifically designed for the study and piloted beforehand on a sample of selected studies. Disagreement was resolved by discussion and consensus with a third reviewer (JHK). The standardized extraction spreadsheet template contained the following data: participant characteristics (percentage male, mean/median age, and OSA prevalence); study characteristics (first author, publication year, study design, study setting, country, and sample sizes for the training, validation, and test datasets where applicable); model characteristics (type of AI classifiers used, feature engineering, method of OSA diagnosis [eg, polysomnography or HSAT], reference standard used for OSA diagnosis, and AHI cutoffs); and the following outcome domains: (1) diagnostic performance of AI models for OSA, including sensitivity, specificity, accuracy, positive predictive value, negative predictive value, AUC; and (2) confusion-matrix components (true positives [TP], true negatives [TN], FP, and false negatives [FN]) where available. In line with PRISMA 2020 expanded checklist, we specified whether all results compatible with each outcome domain were sought. For studies reporting multiple eligible results (ie, several AHI thresholds, different AI model versions, or multiple test sets), we extracted all results that met our predefined inclusion criteria. When confusion matrices were reported, all relevant matrices corresponding to included analyses were extracted. This approach was used to minimize selective-outcome bias across studies.

Where data were missing or unclear, the following assumptions were applied: if sample sizes differed across sections of the manuscript without clarification, we used the number explicitly associated with the relevant dataset (training, validation, or test). When AHI cutoffs were not directly stated, we inferred thresholds based on the authors’ definitions of OSA severity (mild, moderate, or severe), which clearly aligned with standard criteria. If demographic data were reported inconsistently (ie, percentages without denominators), we assumed the total study population as the denominator unless otherwise indicated. We did not impute missing accuracy metrics. No external tool was used to determine which data items to collect.

### Study Risk of Bias Assessment

The quality assessment of included studies was assessed by 2 blinded, independent reviewers (KJMY and CYJL), using the Quality Assessment of Diagnostic Accuracy Studies-2 (QUADAS-2) tool to evaluate the risk of bias and applicability of diagnostic accuracy studies [28]. The QUADAS-2 tool assesses studies on the following 4 key domains, including patient selection, index test, reference standard, and flow and timing. Each domain and the overall study are graded as either low, some concerns, or high risk of bias. If any 1 domain was graded as “some concerns” or “high risk,” the overall risk of bias for that study would be graded as “some concerns” or “high risk,” respectively. Any disputes between reviewers were resolved by consensus from a third independent reviewer (JHK).

### Statistical Analysis, Including Effect Measures, Synthesis Methods, and Reporting Bias Assessment

Binary outcome data (TP, FP, TN, and FN) were used directly from confusion matrices reported in the primary studies. When not directly reported, binary diagnostic accuracy data were derived using the formulas below:

- Sensitivity = TP / (TP + FN)
- Specificity = TN / (TN + FP)
- OSA positive sample size = TP + FN
- OSA negative sample size = TN + FP
- Total sample size = TP + TN + FP + FN
- Accuracy = (TP + TN) / (TP + TN + FP + FN)
- Recall = TP / (TP + FN)

Thus:

- TP = Sensitivity × OSA positive sample size = Recall × OSA positive sample size
- TN = Specificity × OSA negative sample size = Accuracy × Total sample size – TP

When multiple metrics were available, we prioritized directly reported confusion matrices, followed by sensitivity or specificity with prevalence, as these allowed the most accurate reconstruction. Studies for which TP, TN, FP, and FN could not be derived were excluded from the analysis. Minor discrepancies due to rounding were addressed by rounding to the nearest integer, as it is not possible to have less than a whole patient. Further checks were performed by ensuring that the calculated TP, TN, FP, and FN matched the total sample size. As there was no statistical reconstruction but rather simple calculations were performed, there was no statistical uncertainty to account for, and the authors have decided not to perform a meta-regression.

Studies with directly reported or calculated 2×2 data and which evaluated their model with a random split test set or k-fold cross-validation were then pooled in a Bayesian bivariate random effects meta-analysis, using a noninformative prior. As specificity and sensitivity are related, a Bayesian meta-analysis was the chosen method to allow for joint estimation of them. Pooled sensitivity and specificity were summarized using hierarchical summary receiver operating characteristic (HSROC) curves. Diagnostic odds ratio (DOR) and positive and negative likelihood ratios were also derived from the meta-analysis. Between-study heterogeneity was graphically visualized using 95% prediction regions on HSROC curves. Random-effects Bayesian meta-regression was performed for both continuous and categorical study-level covariates, including AI classifiers, feature engineering, AHI cutoffs, sampling frequency, sleep test reference standard, test method, prevalence, age, and gender ratio.

To assess the robustness of the synthesized results, an informative prior (where the lower bound was set as 50% sensitivity/specificity) was applied as a sensitivity analysis for the overall meta-analysis. The potential impact of 4 different mechanisms of publication bias (data, sensitivity, specificity, or DOR-driven) with varying probabilities of unpublished studies (up to 40%) was evaluated via a sensitivity analysis where the HSROC curve, AUC, sensitivity, and specificity were re-estimated for each scenario in a Bayesian hierarchical framework. Risk of bias due to missing results in the synthesis was simultaneously assessed using the same Bayesian selection-model sensitivity analyses. All analyses were conducted following statistical guidance from the Cochrane Handbook and were performed using MetaBayesDTA (1.5.2; University of Leicester) and DTAmetasa (0.9.1; Osaka University) [29-33], built using R (R Foundation for Statistical Computing) and Stan (Stan Development Team) [34-36]. The analyses were performed by one statistician and independently verified by a second analyst. Each AI model was treated as independent observations even if they originated from the same study; AI models were only grouped according to technological factors for the purposes of meta-regression.

For diagnostic accuracy outcomes, the primary effect measures used in the synthesis were sensitivity and specificity, consistent with standard practice for diagnostic test accuracy reviews and Cochrane DTA guidance. Secondary effect measures include DOR, positive likelihood ratio, negative likelihood ratio, and the area under the HSROC curve. No categorical thresholds (ie, “small,” “moderate,” or “large” effects) were applied to interpret sensitivity, specificity, or other accuracy measures; instead, statistical significance and uncertainty were assessed using 95% credible intervals (95% CrI) from the Bayesian models.

### Certainty Assessment/Quality of Evidence

The quality of pooled evidence was evaluated using the Grading of Recommendations Assessment, Development, and Evaluation (GRADE) framework [35]. The GRADE framework rates each study on the basis of study design, consistency, directness, risk of bias, precision, and publication bias. For each outcome, the level of certainty was rated as high, moderate, low, or very low, following standard GRADE decision rules. Evaluations were performed by 2 reviewers (KJMY and CYJL) independently, with disagreements resolved through discussion.

## Results

### Study Selection

**Figure 1.**
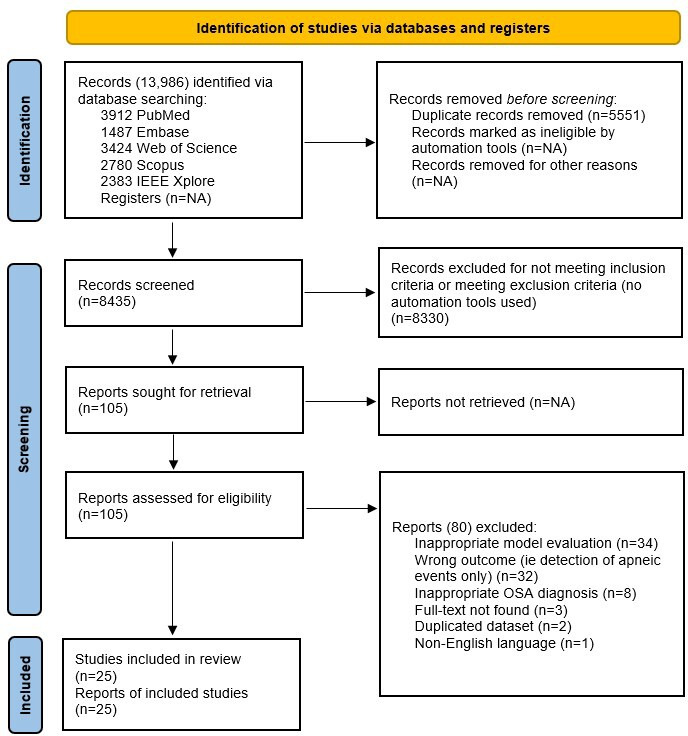
PRISMA (Preferred Reporting Items for Systematic Reviews and Meta-Analyses) flow diagram to summarize the study selection process. NA: not applicable; OSA: obstructive sleep apnea.

A total of 8435 articles were included in the initial search after the removal of duplicates, of which 105 were selected for full-text review based on title and abstract screening. After full-text review, 25 [12,13,20,37-58] articles met the final inclusion criteria and were included in Table S1 in Multimedia Appendix 1. The study selection process is summarized in Figure 1.

Studies that only detected apneic events without a diagnosis of OSA or investigated sleep apnea-hypopnea syndrome but not OSA were excluded [59,60].

### Study Characteristics

**Figure 2.**
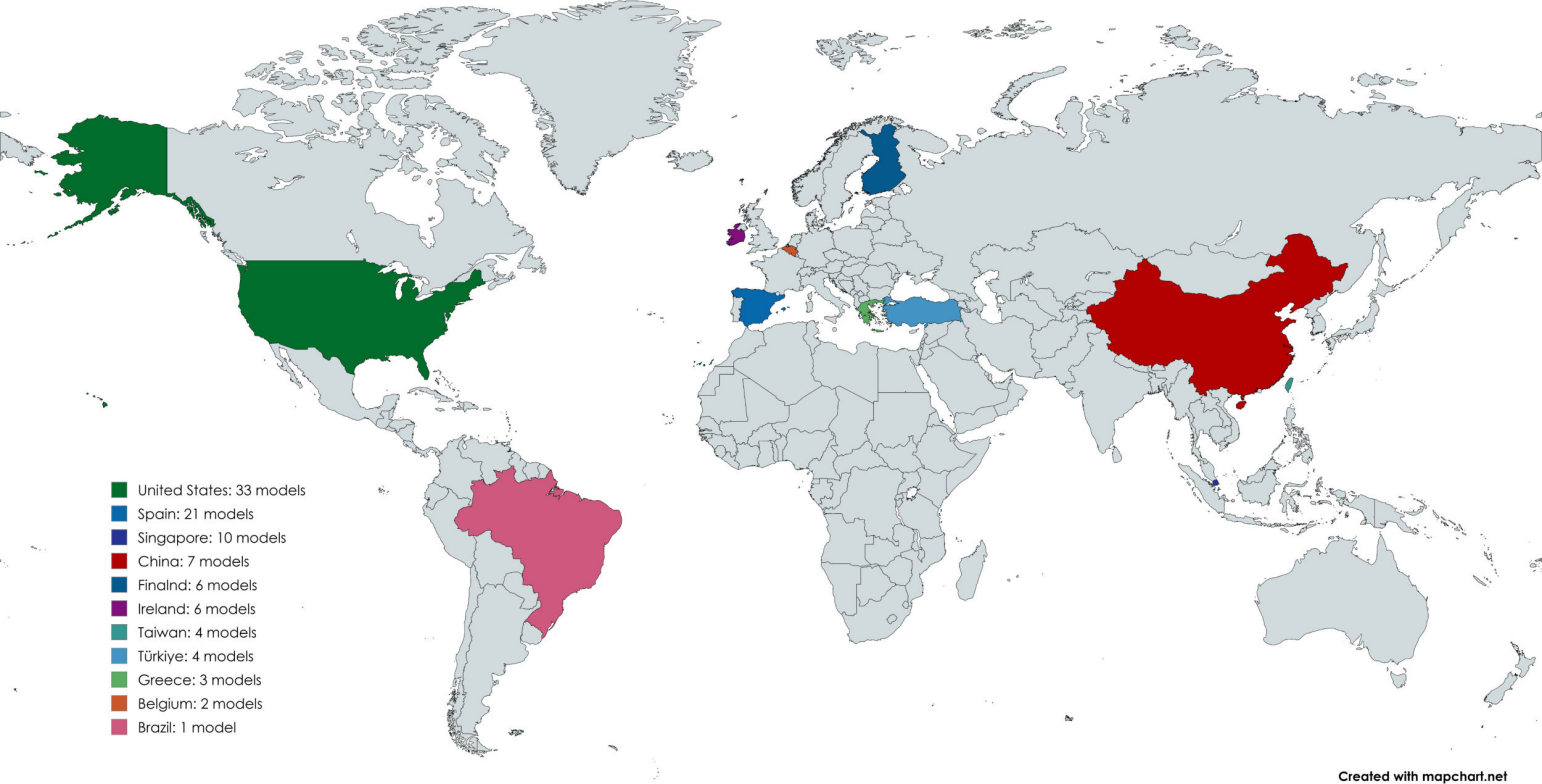
Geographical locations of included studies.

A total of 25 [12,13,20,37-58] cross-sectional studies with 97 AI models were included. As shown in Figure 2, 42 models were tested in European populations, 1 in South America, 33 in North America, and 21 models were tested in an Asian population. The total sample (nonduplicated) consisted of 23,171 participants used to train the AI models and 15,025 participants for testing. Mean age ranged from 40.15 (SD 10.6) to 63.32 (SD 13.3) years, while the BMI of participants ranged from 25.20 to 37.08 kg/m^2^. A summary of the characteristics of each study can be found in Table S1 in Multimedia Appendix 1. There were no studies with a high risk of bias based on QUADAS-2 (Table S2 in Multimedia Appendix 1).

### Results of Individual Studies

Among the 25 studies, 12 [12,13,20,37-41,50,55-57] studies used a Nonin brand of pulse oximeter, within which there were 7 different models; 5 [45-49] studies used the Criticare 504 Oximeter, 1 study each used an Embla N7000 Polysomnography System brand [58], Grael polysomnography [42], SleepSense Adult Soft-Tip SpO_2_ Sensor [51], and Masimo Oximeter [53]. The remaining 4 [43,44,52,54] studies did not specify the brand of pulse oximeter used. As there was a large variety in the brands of pulse oximeters used, the authors have decided not to perform a meta-regression for brand of pulse oximeter. Furthermore, no two studies from independent populations shared the same pulse oximeter brand and model; thus, a meta-regression may not generate any meaningful difference in accuracy. Sampling frequency varied greatly from 0.2 Hz to 500 Hz (Table S3 in Multimedia Appendix 1).

### Reference Standard for OSA Diagnosis

Among the 25 studies, 22 studies [12,13,20,38,39,41-49,51-58] evaluated for OSA using overnight polysomnography, while 3 studies [37,40,50] used HSATs. All studies defined OSA and its severity using the AHI. Among the 97 AI models, 39, 10, 29, and 19 used an AHI cutoff of ≥5, ≥10, ≥15, and ≥30 events/h, respectively, to define the presence of OSA (Table S1 in Multimedia Appendix 1). The prevalence of OSA in the testing set ranged from 12.5% to 93.8% [37,40].

### Artificial Intelligence Models

To extract and select SpO_2_ features, 58 models used deep learning, while 39 models relied on manual feature extraction by a domain expert. To classify the severity of OSA, 15 models used decision trees (comprising 12 classification and regression trees and 3 random forest models), 6 models used linear models (comprising 4 logistic regression and 2 linear discriminant analysis models), 10 models used gradient boost, 46 models used neural networks, and 20 models used support vector machines. A total of 52 models were evaluated with a cross-validation technique (k-fold or leave-one-out) while 45 models were evaluated with a random-split test set.

### Data Presentation

For each study, the raw diagnostic accuracy data (TP, FP, TN, and FN) are presented in Table S4A in Multimedia Appendix 1. These data formed the basis for the hierarchical meta-analysis; individual study-level effect estimates with precision (eg, CIs) were not generated because accuracy estimates were synthesized using the Bayesian hierarchical HSROC framework.

As shown in Table S4A in Multimedia Appendix 1, out of the 25 included studies, 13 studies presented their confusion matrix and allowed for direct extraction of the TP, TN, FP, and FN values. A total of 12 studies required a calculation of the raw accuracy data from OSA prevalence and the presented sensitivity and specificity. Raw data regarding the accuracy, sensitivity, and specificity of each AI model have also been provided.

### Results of Synthesis: Meta-Analysis of Diagnostic Accuracy

All 25 included studies contributed to the primary synthesis of diagnostic accuracy. As summarized in Tables S1, S4A, and S4B in Multimedia Appendix 1, the contributing studies varied in AI classifier type, feature engineering approach, test-set construction methods, and AHI thresholds used for model evaluation. These methodological differences represent important potential sources of heterogeneity when interpreting the pooled results; therefore, subgroup analyses and meta-regressions stratified by these study characteristics were performed. The results of each meta-regression are presented in the sections below. Overall certainty of evidence was rated using the GRADE framework, and the risk of bias was also assessed for all studies using QUADAS-2, with domain-level judgments summarized in the subsequent section.

### Overall Accuracy Statistics

**Figure 3.**
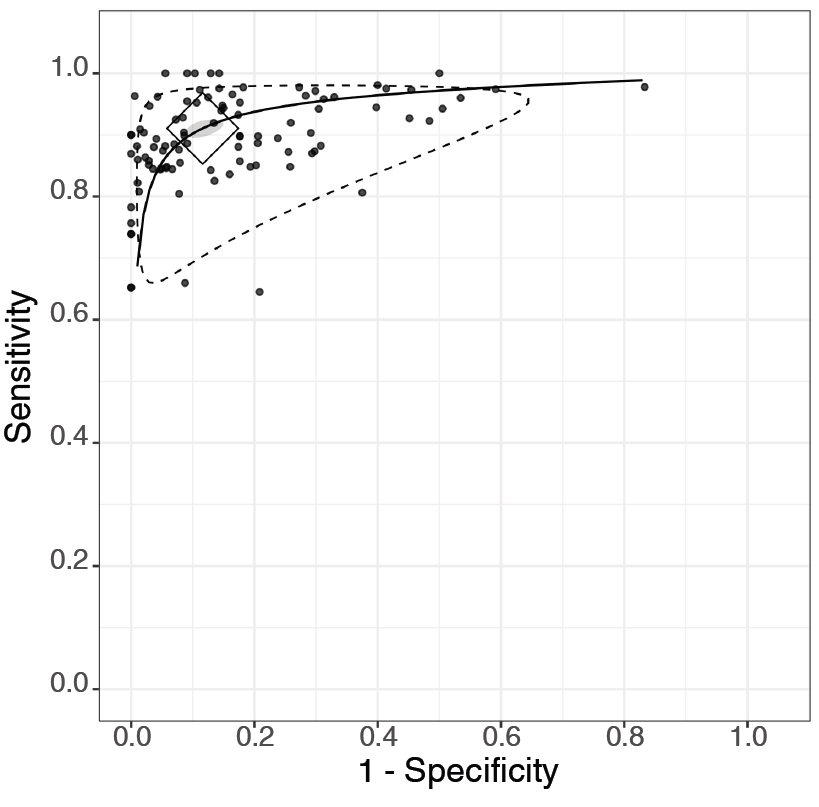
Summary receiver operating characteristic (SROC) plot for the overall obstructive sleep apnea (OSA) diagnostic accuracy of artificial intelligence (AI) models trained on oxygen saturation (SpO_2_) recordings.

Compared to conventional diagnostic methods, the use of AI trained on SpO_2_ recordings achieved a pooled sensitivity of 91.1% (95% CrI 89.7%-92.4%) and specificity of 88.4% (95% CrI 85.3%-90.8%), with a DOR of 77.7 (95% CrI 60.2-99.6), positive likelihood ratio of 7.85 (95% CrI 6.25-9.85), and negative likelihood ratio of 0.10 (95% CrI 0.086-0.116). The summary receiver operating characteristic (SROC) curve is displayed in Figure 3.

A sensitivity analysis using an informative prior yielded identical results, with a sensitivity of 91.1% (95% CrI 89.6%-92.2%) and specificity of 88.4% (95% CrI 85.7%-90.8%), a DOR of 77.3 (95% CrI 61.7-99.0), a positive likelihood ratio of 7.83 (95% CrI 6.40-9.86), and a negative likelihood ratio of 0.10 (95% CrI 0.089-0.117). These minimal differences indicate that the pooled estimates were robust to prior specification.

In Figure 3 the solid line represents the extrapolated SROC curve. The diamond represents the summary receiver operating point. Shaded/dashed regions represent the 95% CrI. Unshaded circles/ovals are centered around individual study means; their height/width are proportionate to study weights for sensitivity/specificity.

### Meta-Regression of AI Classifier

**Figure 4.**
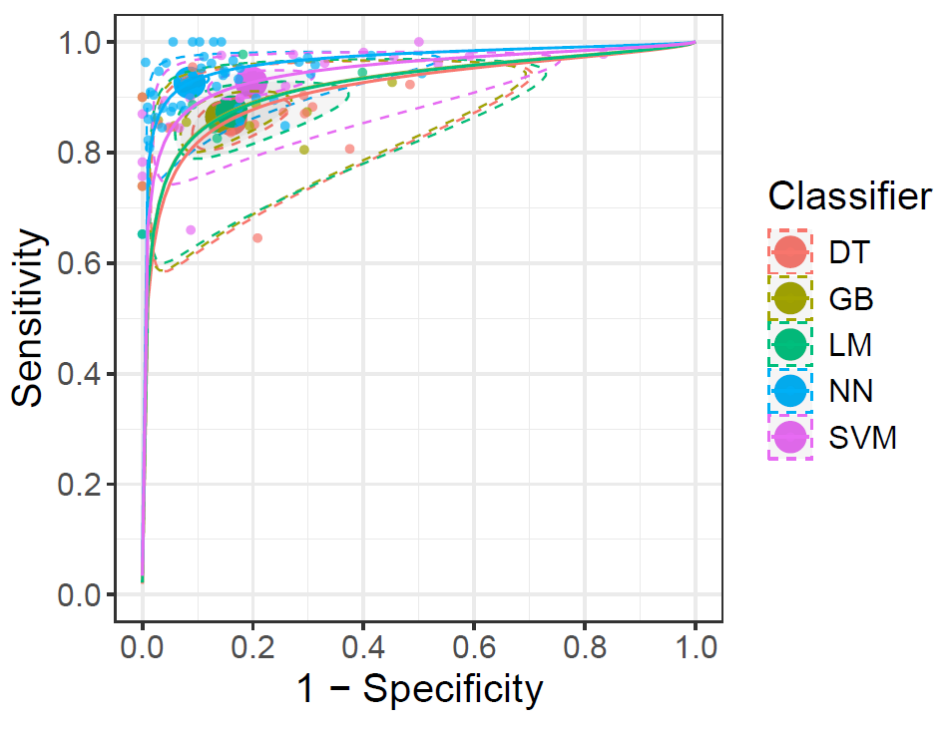
Summary receiver operating characteristic (SROC) plot for Bayesian meta-regression stratified by artificial intelligence (AI) classifier. DT: decision tree; GB: gradient boost; LM: linear model; NN: neural network; SVM: support vector machine.

As shown in Figure S1A in Multimedia Appendix 1, among the different types of AI classifiers involved in this study, neural networks consistently have the highest sensitivity and specificity at 92.7% (95% CrI 91.1%-94.0%) and 91.3% (95% CrI 87.9%-93.5%), respectively. The sensitivity of neural networks is significantly higher than that of decision trees (difference in sensitivity: –6.7%, 95% CrI –11.9% to –2.4%) and gradient boost (difference in sensitivity: –6.3%, 95% CrI –12.6% to –1.8%). There was no significant difference in sensitivity when compared to linear models. There were no significant differences in specificities among neural networks, gradient boosts, linear models, and decision trees. The next best model is a support vector machine with a sensitivity of 92.7% (95% CrI 89.8%-94.8%) and a specificity of 80.0% (95% CrI 70.1%-87.4%). There was no significant difference in sensitivity (95% CrI –2.6 to 3.1%) between the support vector machine and the neural network. However, the neural network was more specific (difference in specificity: 11.2%, 95% CrI 3.30%-21.1%) than the support vector machine. The remaining models are comparable in sensitivity and specificity. Visual comparison of the SROC curves corroborates the above findings, with the neural network having the best performance, followed by the support vector machine, with the remaining 3 models being comparable, as shown in Figure 4.

In Figure 4 the solid lines represent the extrapolated SROC curves. Large circles represent the summary operating points. The shaded areas represent the 95% CrI, and the dotted lines represent the 95% prediction region. Small circles represent individual study estimates.

**Figure 5.**
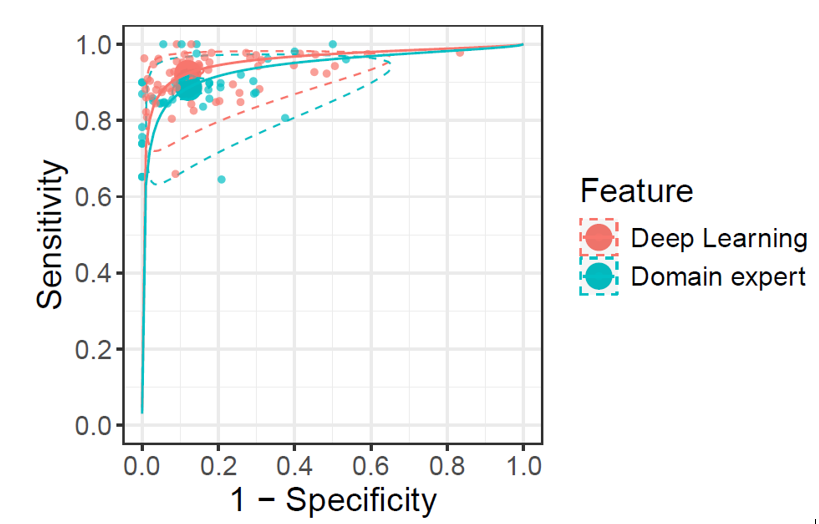
Summary receiver operating characteristic (SROC) plot for Bayesian meta-regression stratified by type of artificial intelligence feature engineering. Solid lines represent the extrapolated SROC curves. Large circles represent the summary operating points. The shaded areas represent the 95% credible regions, and the dotted lines represent the 95% prediction region. Small circles represent individual study estimates.

In addition, for feature extraction and selection, deep learning (92.3%, 95% CrI 90.8%-93.6%) displayed significantly higher sensitivity as compared to domain expert (88.6%, 95% CrI 85.8%-90.9%) with a difference of 3.7% (95% CrI 0.9%-6.9%; Figure S1B in Multimedia Appendix 1). However, there were no significant differences in specificities. The SROC curves can be found in Figure 5.

### Meta-Regression of AHI

At the clinically relevant AHI cutoffs of ≥5, ≥10, ≥15, and ≥30, the sensitivities were 93.4% (95% CrI 91.6%-94.8%), 91.3% (95% CrI 86.4%-94.7%), 88.8% (95% CrI 85.6%-91.2%), and 88.1% (95% CrI 83.7%-91.1%), respectively, while the specificities were 79.0% (95% CrI 72.3%-85.1%), 88.6% (95% CrI 79.2%-93.9%), 87.9% (95% CrI 83.5%-91.6%), and 95.7% (95% CrI 93.1%-97.3%). These data are visualized in Figure S1C in Multimedia Appendix 1.

Sensitivity was the highest at lower AHI cutoffs, while specificity increased as the AHI cutoff increased. While an AHI cutoff of ≥5 had no significantly different sensitivity than an AHI cutoff of ≥10 (difference 2.0%, 95% CrI –1.6% to 7.0%), it had a significantly higher sensitivity than an AHI cutoff of ≥15 (difference 4.5%, 95% CrI 1.5%-8.2%) and ≥30 (difference 5.3%, 95% CrI 1.8%-9.9%).

An AHI cutoff of ≥5 has a significantly lower specificity than an AHI cutoff of ≥30 with a difference of –16.6% (95% CrI –23.9% to –10.3%), and an AHI cutoff of ≥15 has a significantly lower specificity than an AHI cutoff of ≥30 with a difference of –7.7% (95% CrI –12.1% to –3.1%).

**Figure 6.**
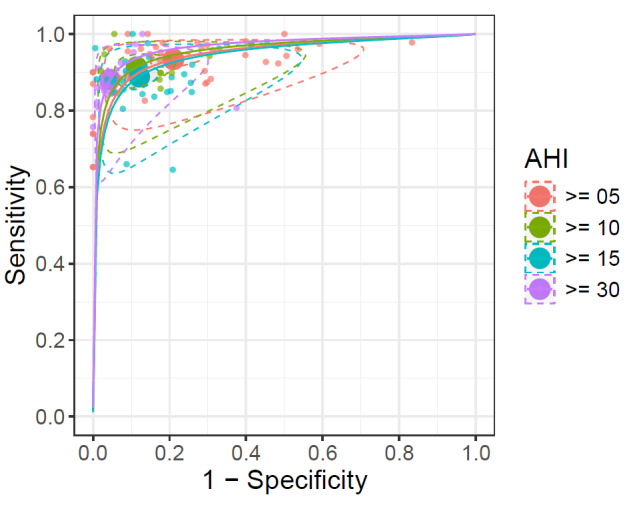
Summary receiver operating characteristic (SROC) plot for Bayesian meta-regression stratified by apnea-hypopnea index (AHI) cutoffs of ≥5, ≥10, ≥15, and ≥30. Solid lines represent the extrapolated SROC curves. Large circles represent the summary operating points. The shaded areas represent the 95% credible regions, and the dotted lines represent the 95% prediction region. Small circles represent individual study estimates.

Other statistics are summarized in Table 1. Visual comparison of the SROC curves for each AHI cutoff suggested that performance was essentially similar across the various AHI cutoffs, as the curves were closely overlapping. The differences in sensitivity and specificity appeared to be mainly due to threshold shifts along the SROC curve, rather than shifts of the entire SROC curve Figure 6.

**Table 1.** Summary of diagnostic test accuracy statistics from Bayesian meta-analysis at clinically relevant thresholds.

Subgroup	Posterior, median (95% posterior interval)					
	Sensitivity	Specificity	FPR^a^	DOR^b^	LR+^c^	LR–^d^
Overall	91.1 (89.7-92.4)	88.4 (85.3-90.8)	11.6 (9.2-14.7)	77.7 (60.2-99.6)	7.85 (6.25-9.85)	0.10 (0.09-0.12)
AHI^e^≥5	93.4 (91.6-94.8)	79.0 (72.3-85.1)	21.0 (14.9-27.7)	52.7 (36.7-80.5)	4.44 (3.39-6.22)	0.085 (0.07-0.11)
AHI≥10	91.3 (86.4-94.7)	88.6 (79.2-93.9)	11.4 (6.1-20.8)	81.8 (39.5-173.4)	8.00 (4.47-14.81)	0.10 (0.06-0.15)
AHI≥15	88.8 (85.6-91.2)	87.9 (83.5-91.2)	12.1 (8.4-16.5)	58.2 (39.6-84.9)	7.35 (5.45-10.48)	0.13 (0.10-0.16)
AHI≥30	88.1 (83.7-91.1)	95.7 (93.1-97.3)	4.3 (2.7-6.9)	160.7 (100.5-267.7)	20.3 (12.82-31.12)	0.125 (0.094-0.169)

a FPR: false positive rate (1 – specificity).

b DOR: diagnostic odds ratio.

c LR+: likelihood ratio positive.

d LR–: likelihood ratio negative.

e AHI: apnea-hypopnea index.

### Meta-Regression of Test Method

To address the methodological heterogeneity introduced by different test methods, a meta-regression comparing studies that used random-split tests versus those that used k-fold cross-validation was conducted. Models evaluated using random-split test sets demonstrated a pooled sensitivity of 88.6% (95% CrI 85.8%-90.9%) and specificity of 87.8% (95% CrI 82.7%-92.0%), whereas models assessed with cross-validation showed a pooled sensitivity of 92.3% (95% CrI 90.8%-93.6%) and specificity of 88.0% (95% CrI 84.4%-91.2%).

**Figure 7.**
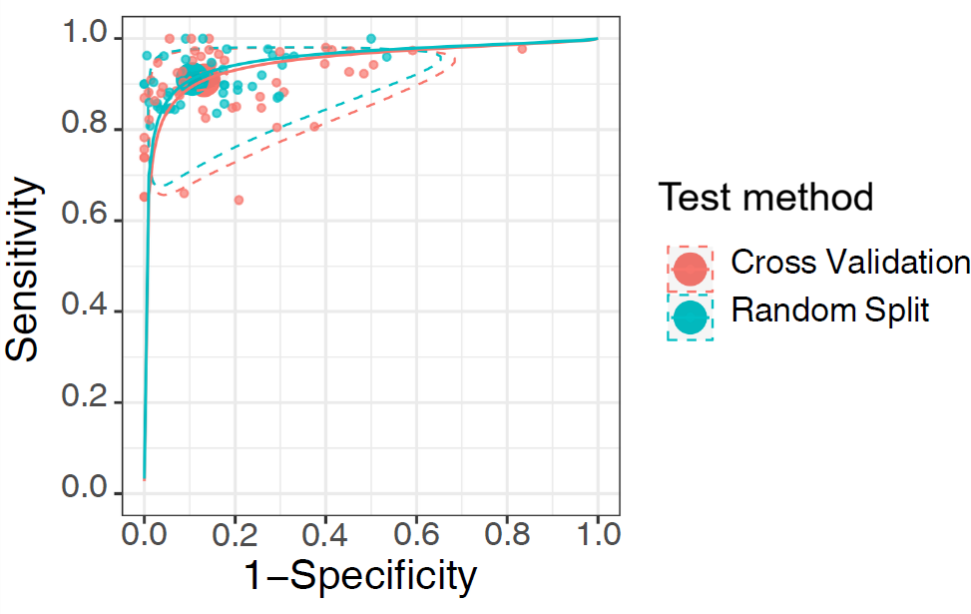
Summary receiver operating characteristic (SROC) plot for Bayesian meta-regression stratified by test method.

The meta-regression confirmed that cross-validation was more sensitive (difference 3.7%, 95% CrI 0.9%-6.9%) but not significantly more specific (95% CrI –5.5% to 6.4%) than random split (Figure S1D in Multimedia Appendix 1). The corresponding SROC curves can be found in Figure 7. These findings indicate that differences in test method could have potentially affected diagnostic accuracy.

In Figure 7 solid lines represent the extrapolated SROC curves. Large circles represent the summary operating points. The shaded areas represent the 95% credible regions, and the dotted lines represent the 95% prediction region. Small circles represent individual study estimates.

### Meta-Regression of Prevalence

Sensitivity remained consistent at 91.1% (95% CrI 89.7%-92.4%) despite changes in percentage prevalence, though specificity decreased as percentage prevalence increased, with an average of 86.7% (95% CrI 84.5%-88.7%; Figure S2A in Multimedia Appendix 1).

### Meta-Regression of Age and Gender

Conversely, average age and the percentage of male participants were not associated with sensitivity or specificity (Figure S2B and S2C in Multimedia Appendix 1).

### Meta-Regression of Sampling Frequency

Sensitivity remained constant at 91.1% (95% CrI 89.7%-92.4%), while specificity decreased as sampling frequency increased, with an average of 88.0% (95% CrI 85.0%-90.8%; Figure S3A in Multimedia Appendix 1).

### Meta-Regression of Reference Standard

**Figure 8.**
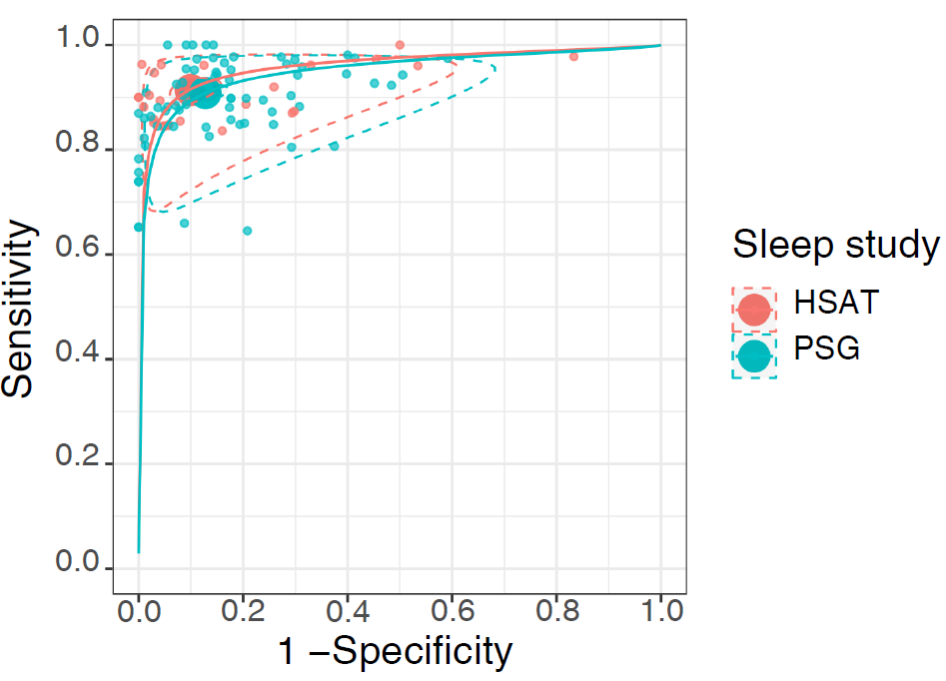
Summary receiver operating characteristic (SROC) for Bayesian meta-regression stratified by reference standard. Solid lines represent the extrapolated SROC curves. Large circles represent the summary operating points. The shaded areas represent the 95% credible regions, and the dotted lines represent the 95% prediction region. Small circles represent individual study estimates. HSAT: home sleep apnea test; PSG: polysomnography.

To determine if using polysomnography versus HSAT as the reference standard contributed to the heterogeneity between studies, a meta-regression was performed. Studies using polysomnography achieved a pooled sensitivity and specificity of 90.8% (95% CrI 89.2%-92.3%) and 87.4% (95% CrI 83.2%-90.5%), respectively (Figure 8). Studies using HSAT achieved a pooled sensitivity and specificity of 91.5% (95% CrI 89.0%-93.7%) and 90.0% (95% CrI 85.0%-93.5%). Meta-regression showed that this difference is not statistically significant, with the difference in sensitivity being 0.7% (95% CrI –2.4% to 3.4%) and the specificity at 2.5% (95% CrI –3.3% to 7.4%; Figure S3B in Multimedia Appendix 1).

### Sensitivity Analysis and Restricting Datasets

Several studies trained and tested their AI models on the same publicly available dataset, introducing nonindependence and potentially affecting the overall diagnostic accuracy. Furthermore, this overlap in datasets could decrease study heterogeneity and generalizability. A total of 5 studies shared the Sleep Heart Health Study (SHHS 1 and 2) datasets, 4 studies shared the Clínico de Santiago de Compostela, Spain dataset, 2 studies shared the Multiethnic Study of Atherosclerosis and the Osteoporotic Fractures in Men Study datasets, while 2 studies shared the University College of Dublin dataset (Table S4B in Multimedia Appendix 1).

To maintain rigor and ensure validity of our meta-analytic results, a sensitivity analysis was done, ensuring that only one study per dataset was performed. To decide which study to exclude in the sensitivity analysis, we used the following criteria in decreasing order of priority:

1. Availability of an external, independent testing set (highest priority)
2. Largest test set
3. Publication date (lowest priority)

Availability of an external, independent test set was allocated the highest priority, as it directly evaluates the AI model in an independent patient population, restoring study independence. Test set size was then considered to help reduce random error. Finally, publication date was considered to ensure results reported were the latest and most up-to-date. Of the 11 studies containing shared datasets, 3 [49,54,56] of them were selected to be included in the sensitivity analysis, while the other 8 [20,40,44-48,55,57] were excluded.

**Figure 9.**
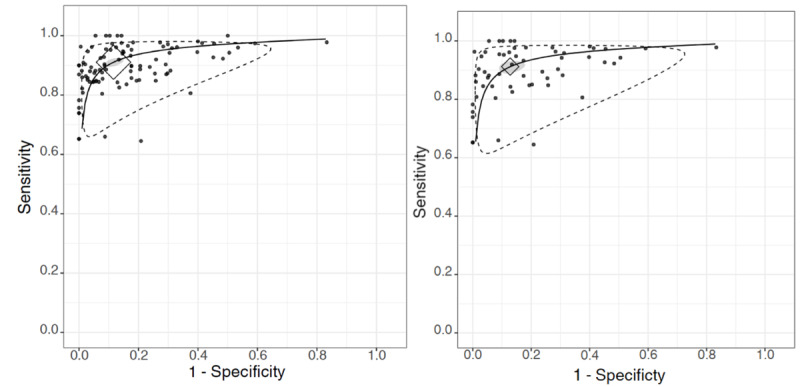
Original summary receiver operating characteristic (SROC) plot for the overall obstructive sleep apnea (OSA) diagnostic accuracy of artificial intelligence (AI) models trained on oxygen saturation (SpO_2_) recordings (left) versus after excluding studies with duplicate datasets (right).

After ensuring that each study had a unique dataset for training and testing, the pooled sensitivity and specificity had changed minimally. The sensitivity after exclusion was 91.2% (95% CrI 89.1%-93.1%) while specificity was 87.2% (95% CrI 82.4%-90.8%). This demonstrated that the sharing of datasets had minimal effect on the pooled sensitivity and specificity. Visual comparison of the SROC curves also depicts minimal shifts (Figure 9).

In Figure 9, the solid line represents the extrapolated SROC curve. The diamond represents the summary receiver operating point. Shaded/dashed regions represent the 95% CrI or prediction intervals. Unshaded circles/ovals are centered around individual study means; their height/width are proportionate to study weights for sensitivity/specificity.

### Reporting Biases

#### Publication Bias

**Figure 10.**
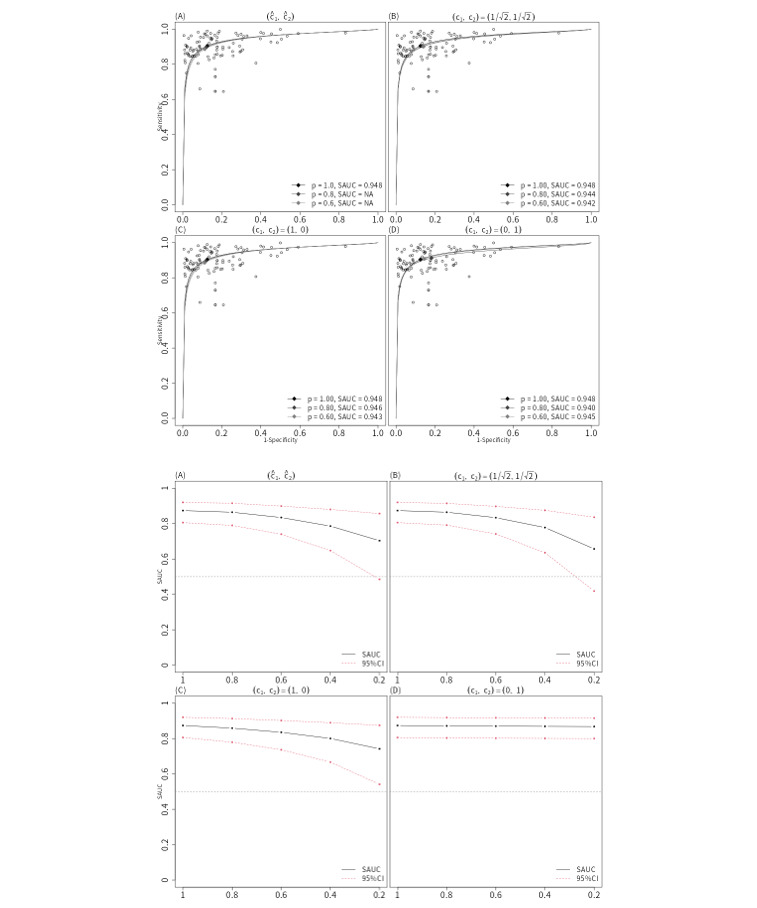
Effect of varying scenarios of publication bias on the (1) summary receiver operating characteristic (SROC) curve (parts A to D in top half of the figure) and (2) area under the curve (AUC) (parts A to D in the bottom half of the figure). SAUC: summary AUC.

Overall, the risk of bias due to missing results is judged to be low, as sensitivity analyses on the SROC curve and AUC suggested no clinically significant publication bias. When considering 4 different mechanisms of publication bias (data, sensitivity, specificity, or DOR-driven), with varying probabilities of unpublished studies (up to 40%), the SROC curve (Figure 10) and AUC (Figure 10) were almost constant, where the AUC shifted only modestly (0.902-0.877). This suggests that even if most studies remained unpublished, the conclusions of this meta-analysis would not have changed. For data-driven bias, sensitivity decreased from 81.5% to 78.7% and specificity from 86.1% to 84.1%. For sensitivity-driven bias, sensitivity declined from 81.5% to 78.6% and specificity from 86.1% to 84.2%. For specificity-driven bias, sensitivity declined from 81.5% to 74.4%, while specificity slightly increased from 86.1% to 87.3%. For DOR-driven bias, sensitivity rose slightly (81.5%-83.6%) but specificity fell (86.1%-78.2%). These shifts indicate that even under extreme assumptions of unpublished data, the summary estimates changed only modestly, and the overall conclusions of the meta-analysis remained unchanged.

In Figure 10, the potential impact of 4 different mechanisms of publication bias is shown: (A) data-driven, (B) sensitivity-driven, (C) specificity-driven, and (D) DOR-driven, with varying probabilities of unpublished studies (0%, 20%, and 40%). The solid lines and diamonds represent the SROC curves and summary operating points. The summary AUC denotes the area under the SROC curve. Black and red dots represent the mean and 95% CIs of the AUC for each probability of unpublished studies (x-axis).

#### Risk of Bias in Studies

**Figure 11.**
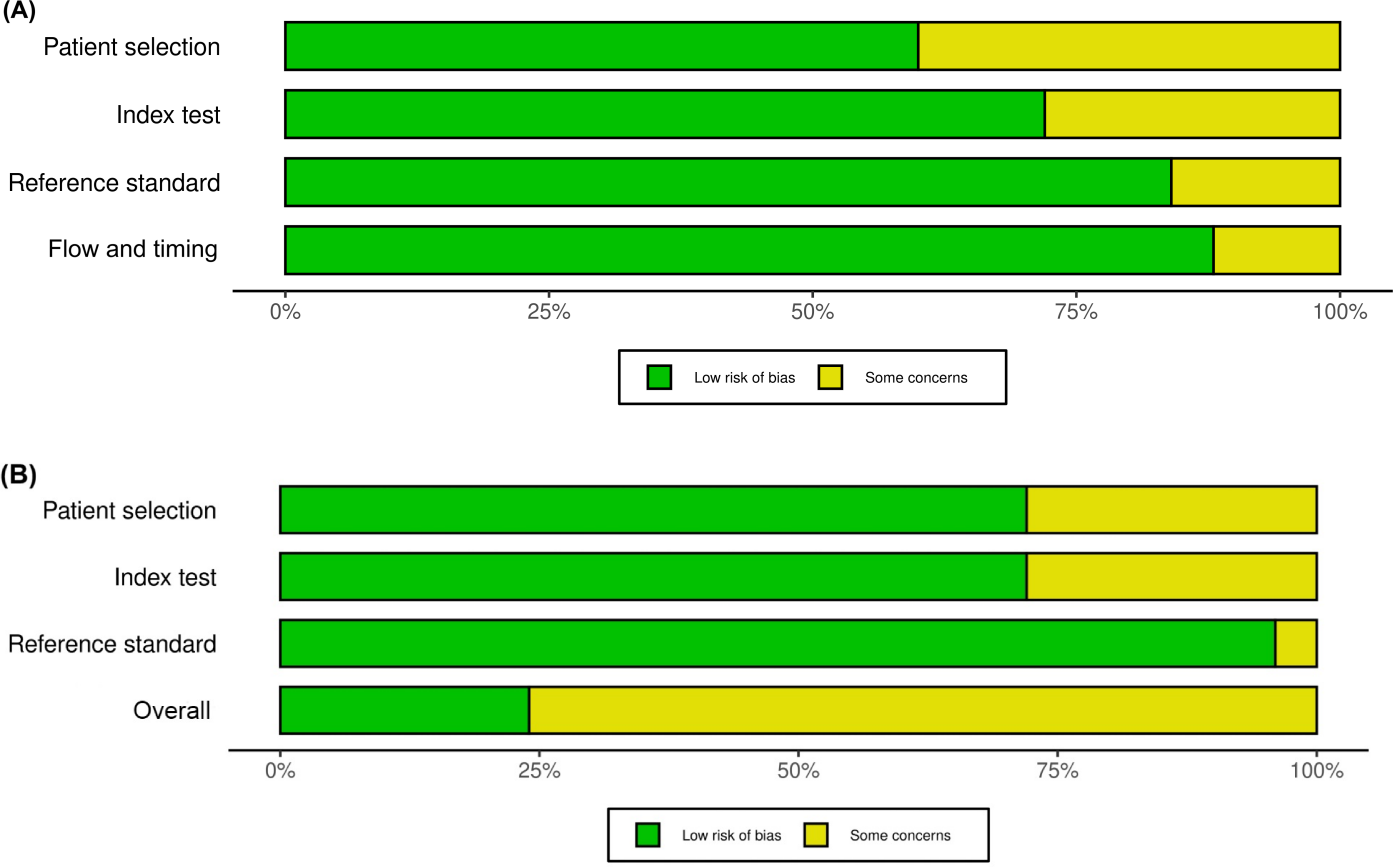
Quality Assessment of Diagnostic Accuracy Studies-2 (QUADAS-2) visualization tool for (A) risk of bias assessment and (B) concerns regarding applicability.

Using QUADAS-2 to evaluate the risk of bias among our included studies demonstrated that within each domain, most studies had a low risk of bias. Details of the assessment are provided in Table S2 in Multimedia Appendix 1. The proportions of low-risk studies for patient selection, index test, reference standard, and flow and timing were 60%, 72%, 84%, and 84%, respectively (Figure 11A). Regarding applicability, 72%, 72%, and 96% of studies were scored as low risk for patient selection, index test, and reference standard domain (Figure 11B). Overall, these findings indicate that the included studies generally had low risk of bias and good applicability to the review question.

### Certainty of Evidence/Quality of Evidence

The quality of evidence at the outcome level is summarized in Table 2. The overall quality of evidence was high. There was clear evidence of a sensitivity-specificity relationship.

Overall, the results should be interpreted cautiously due to the observational nature of between-study comparisons and the potential for residual confounding from unmeasured study-level factors. Although QUADAS-2 assessments showed that most studies had low risk of bias across major domains, issues in patient selection were more common, reflecting the use of retrospective or convenience samples in several studies. Such sampling approaches may limit representativeness relative to real-world screening populations. Nonetheless, extensive publication-bias analyses demonstrated that even under extreme assumptions of missing studies, the SROC curve and AUC shifted only minimally, supporting the robustness of the main conclusions. However, as the random-split test set subgroup showed marginally lower accuracy, the quality of evidence according to the GRADE framework is only moderate, as external validation is still required to reliably evaluate model performance in new populations and settings.

**Table 2.** Evaluation of quality of pooled evidence using the Grading of Recommendations Assessment, Development, and Evaluation (GRADE) framework (sensitivity: 0.91, 95% CI 0.90-0.92; specificity: 0.88, 95% CI 0.85-0.91; prevalences examined: 15%, 30%, and 60%). The question was as follows: what is the accuracy of artificial intelligence trained on overnight SpO_2_^a^ recordings for diagnosing OSA^b^ in adults?

Outcome	Number of studies; number of patients	Study design	Factors that may decrease certainty of evidence					Effect per 1000 patients tested			Test accuracy CoE^c^
			Risk of bias	Indirectness	Inconsistency	Imprecision	Publication bias	Pretest probability of 15%, mean (95% CI)	Pretest probability of 30%, mean (range)	Pretest probability of 60%, mean (range)	
True positives (patients with OSA) False negatives (patients incorrectly classified as not having OSA)	25 [12,13,20,37-58] studies; 23,171 patients	Cross-sectional (cohort type accuracy study)	Serious	Not serious	Not serious	Not serious	Dose response gradient	137 (135-139) 13 (11-15)	27 (23-31) 273 (269-277)	547 (539-554) 53 (46-61)	⨁⨁⨁⨁^d^ High
True negatives (patients without OSA) False positives (patients incorrectly classified as having OSA)	25 [12,13,20,37-58] studies; 23,171 patients	Cross-sectional (cohort type accuracy study)	Serious	Not serious	Not serious	Not serious	Dose response gradient	751 (725-772) 99 (78-125)	619 (597-636) 81 (64-103)	354 (341-363) 46 (37-59)	⨁⨁⨁⨁ High

a SpO_2_: oxygen saturation.

b OSA: obstructive sleep apnea.

c CoE: certainty of evidence.

d Grading of Recommendations Assessment, Development, and Evaluation. The four symbols (⊕⊕⊕⊕) indicate high certainty of evidence and are a standard component of GRADE assessment tables.

## Discussion

### Principal Findings

This study quantitatively pooled the diagnostic performance metrics of 97 AI models in diagnosing OSA using pulse oximeter readings and explored factors influencing accuracy via meta-regression. Overall, AI models demonstrated high accuracy that could rival polysomnography alternatives. Neural network classifiers were associated with the greatest sensitivity and specificity, while models using deep learning for feature extraction outperformed those relying on domain expert features. Models had higher specificity when predicting more severe OSA, and sensitivity remained high at low AHI thresholds. There was no evidence to suggest publication bias.

To our knowledge, this is the first review to estimate the pooled diagnostic accuracy of AI models trained on oximetry data. Prior reviews were largely narrative, broader in scope, or focused on other input features such as facial features, speech, or heart rate [61-64]. This review advances the field by offering a clearer and more reliable evidence base to support the potential of oximetry-based AI as a scalable tool for OSA screening and diagnosis. Potential uses for such AI models include the rapid and convenient diagnosis of OSA not just in the primary health care setting but also in inpatients who are admitted for complications known to be associated with OSA, such as acute myocardial infarctions, strokes, or arrhythmias like atrial fibrillation [65]. This could potentially facilitate earlier diagnosis of OSA and thus earlier treatment, preventing further complications of the disease [66].

The pooled sensitivity and specificity of AI oximetry models are comparable to or exceeding existing polysomnography alternatives such as ODI and HSAT. Notably, AI approaches appear to outperform the use of ODI alone, which is often only one feature among several used to train these models. For example, Marcos et al [45] reported that a neural network achieved 31% higher sensitivity than ODI3, with only a modest 12% reduction in specificity. Commercial oximetry devices such as the Wellue O₂ Ring and Samsung Galaxy Watch 4 demonstrate lower diagnostic performance, with reported sensitivity and specificity of 87% and 78% (ODI 11) and 90% and 64% (ODI 5), respectively, at their optimal thresholds [67,68]. In contrast, this meta-analysis found higher pooled sensitivity (91.1%) and specificity (88.4%) for AI-driven oximetry. Compared with photoplethysmography-based HSAT devices such as WatchPAT (Itamar Medical Ltd), AI oximetry also performed better at lower AHI cutoffs, with higher specificity and similar sensitivity [69]. These findings suggest that AI oximetry may improve diagnostic performance over traditional ODI and HSAT metrics. However, whether this translates into real-world clinical benefit remains uncertain, and prospective studies are needed to confirm accuracy, usability, and integration into clinical workflows outside research settings.

AI models outperform ODI likely because they incorporate richer temporal and morphological information from SpO_₂_ signals, while deep learning approaches further enhance performance beyond traditional machine learning. The ODI may fail to detect apneas that do not lead to desaturations beyond the predefined threshold [70]. In contrast, AI models may capture additional pulse oximetry features, often using dynamic or differential desaturation thresholds across the recording to capture nonlinear patterns [71]. The slope, shape, interval, and depth of desaturation can also be incorporated, unlike in ODI. Even among the AI models, the superior sensitivity and specificity of neural network classifiers may be attributed to deep learning’s ability to automatically learn intricate patterns from raw data through multiple representation layers, surpassing traditional machine learning approaches [72]. This enables extraction of higher-level features and identification of complex desaturation patterns over extended signal segments, which may even be imperceptible to humans [73-75]. Together, these mechanisms explain both the advantage of AI over ODI and the additional performance gains achieved by deep learning over conventional machine learning approaches.

The lower diagnostic accuracy of a random-split test model could perhaps be explained by the use of external validation datasets. The studies by Gutierrez-Tobal et al [40] and Nikkonen et al [50] were grouped under random-split for meta-regression purposes. However, unlike the other studies that used internal validation, they implemented an external dataset for testing that was not used for training at all. Lower accuracy is expected when models are tested on external datasets from different populations, although this is likely more representative of real-world performance across heterogeneous settings. This helps to prevent overfitting, which may be associated with machine learning models using internal validation, generating overly optimistic accuracy data [76,77]. The predominance of internal validation in included studies highlights the need for more external validation to strengthen generalizability in OSA diagnosis.

The strengths of our study include the use of state-of-the-art Bayesian bivariate random-effects meta-analytic methods, allowing joint estimation of sensitivity and specificity while accounting for between-study variability. This approach supports precise and robust pooled estimates of diagnostic accuracy. Sensitivity analyses using selection models showed that even under extreme assumptions of publication bias, changes in AUC, sensitivity, and specificity were modest, and the overall conclusions remained unchanged. Risk of bias (QUADAS-2) assessment indicated that most studies were at low risk across domains, with generally limited applicability concerns, while overall quality of evidence (GRADE) was high. Taken together, these findings support the robustness and reliability of the pooled estimates.

Nonetheless, several limitations of this study should be considered. These can be broadly grouped into issues affecting internal validity and external validity. Limitations related to internal validity include challenges inherent to the AI diagnostic literature and meta-analytic modeling. Unlike conventional clinical studies, AI models often report only the best-performing configuration among multiple internally tested variants, with selective emphasis on favorable thresholds, architectures, or preprocessing pipelines [78,79]. Consequently, although our publication-bias sensitivity analyses using selection models did not identify substantial evidence of bias, the ability of traditional frameworks to detect selective reporting in AI diagnostic research remains limited [80]. In addition, the retrospective nature of most included studies may introduce bias in data collection and model evaluation, as model development and testing were conducted on preexisting datasets rather than prospectively collected data. Device-level variability (eg, oximeter type, sampling rate, and signal processing) also contributed to heterogeneity [81], since the included studies used 6 different brands of pulse oximeters, and one-third of studies did not report the brand. Several studies contributed multiple models or thresholds, which potentially introduced within-study correlation that could not be fully accounted for within the present modeling package. The potential for overestimated precision should thus be considered when interpreting the results. Furthermore, formal quantification of the proportion of variability due to statistical heterogeneity (ie, *I*²) was not available in the current Bayesian statistical package [82], although visualization of prediction intervals and meta-regression suggested broadly consistent effects.

Limitations affecting external validity relate primarily to generalizability to routine clinical practice. Pulse oximetry is subject to known physiological and technical limitations, including motion artifact, probe displacement, and interference from factors such as skin pigmentation, nail varnish, dirt, temperature, anemia, and hemoglobinopathies [83,84]. In addition, it cannot directly detect cortical arousals or reliably distinguish obstructive from central apneas, which may lead to misclassification or underestimation of disease severity [85]. Generalizability is limited by the predominance of sleep clinic and hospital-based cohorts with high pretest probability of OSA, which reduces applicability to low-prevalence settings such as primary care. Although meta-regression suggested relatively stable sensitivity and improved specificity in lower-prevalence contexts, these findings should be interpreted cautiously given the retrospective nature of all included studies. The absence of prospective real-world validation further limits the ability to draw firm conclusions about clinical readiness, particularly in primary care or acute care environments where case mix, noise, and workflow constraints differ substantially from research settings. Additional limitations include exclusion of non-English studies, which may introduce selection bias [86], and the use of continent of study as a proxy for ethnicity due to missing individual-level data, which may mask important interethnic differences in SpO_₂_ accuracy [87]. Underrepresentation of certain regions, particularly Africa and South America, further limits global generalizability. Finally, reliance on publicly available datasets may introduce regional over-representation, limiting transferability across diverse health care systems.

Future studies evaluating the potential of AI for OSA diagnosis should adopt prospective study designs, with control for pulse oximeter brands and inclusion of general population cohorts encompassing both obstructive and central sleep apnea. Greater representation from underrepresented regions such as South America and Africa is also needed, alongside dedicated studies in pediatric populations where diagnosis remains challenging due to the need for hospitalization and complex equipment. Further work should also explore the role of AI in complementing polysomnography for risk stratification; for example, by leveraging high sensitivity at low AHI thresholds to rule out OSA and high specificity at higher thresholds to support case identification.

### Conclusion

AI-oximetry models showed high diagnostic accuracy for OSA across models and AHI cutoffs, performing better than or comparably to traditional overnight oximetry and HSATs. This review is innovative because it provides the first pooled quantitative synthesis of AI models trained solely on oximetry data, with additional evaluation of publication bias and methodological limitations. Unlike prior reviews, which are largely narrative or broader in scope and include heterogeneous AI inputs such as polysomnography-derived features or multimodal data, this review focuses specifically on pooled diagnostic accuracy and publication bias in AI-oximetry studies. It advances the field by offering a clearer and more reliable evidence base on AI-oximetry performance. These findings support the potential of oximetry-based AI as a scalable, low-cost screening tool for OSA, with potential real-world applications in both primary care and inpatient settings for early identification of high-risk patients. Prospective external validation in diverse populations and low-prevalence settings is still needed before widespread real-world use.

## Supplementary material

10.2196/80349 Multimedia Appendix 1 Overnight pulse oximetry for artificial intelligence diagnosis of obstructive sleep apnea: a Bayesian meta-analysis (online supplement).

10.2196/80349 Checklist 1 PRISMA 2020 for Abstracts checklist.

10.2196/80349 Checklist 2 PRISMA-S checklist.

10.2196/80349 Checklist 3 PRISMA Expanded checklist.

## Abbreviations

AHI: apnea-hypopnea index
AI: artificial intelligence
AUC: area under the curve
CrI: credible interval
DOR: diagnostic odds ratio
FN: false negatives
FP: false positives
GRADE: Grading of Recommendations Assessment, Development, and Evaluation
HSAT: home sleep apnea test
HSROC: hierarchical summary receiver operating characteristic
ODI: oxygen desaturation index
OSA: obstructive sleep apnea
PRISMA: Preferred Reporting Items for Systematic Reviews and Meta-Analyses
PRISMA-S: Preferred Reporting Items for Systematic Reviews and Meta-Analyses Literature Search Extension
PROSPERO: International Prospective Register of Systematic Reviews
QUADAS-2: Quality Assessment of Diagnostic Accuracy Studies-2
SHHS: Sleep Heart Health Study
SpO_2_: oxygen saturation
SROC: summary receiver operating characteristic
STOP-BANG: Snoring, Tiredness, Observed apnoea, Pressure (hypertension), BMI>35 kg/m^2^, Age>50, Neck circumference>40 cm, and Gender (male)
TN: true negatives
TP: true positives
