# Supplementary material for: Artificial Intelligence Diagnosis of Obstructive Sleep Apnea Using Overnight Pulse Oximetry: A Systematic Review and Bayesian Meta-Analysis
Source: J Med Internet Res. 2026 Jul 8;28:e80349. doi: 10.2196/80349 (PMC13344538; doi:10.2196/80349)
Supplement: Multimedia Appendix 1 [file jmir-v28-e80349-s001.docx]

**Overnight Pulse Oximetry for Artificial Intelligence Diagnosis of Obstructive Sleep Apnoea: A Bayesian Meta-Analysis (Online supplement)**

Kvan Jie Ming Yam, Claire Yi Jia Lim, Esther Yanxin Gao, MBBS, MRCS, Jin Hean Koh, MBBS, Nicole Kye Wen Tan, MBBS, Adele Chin Wei Ng, MBBS, MMed (ORL), Zhou Hao Leong, MBBS, MMed (ORL), Chu Qin Phua, MBChB, MMed (ORL), FRCS, Thun How Ong, MBBS, MRCP, Leong Chai Leow, MD, FRACP, FAMS, Guang-Bin Huang, PhD, Benjamin Kye Jyn Tan, MBBS (Hons), MRCS, Song Tar Toh, MBBS, MMed (ORL), MMed (Sleep Med), FAMS (ORL)

[SUPPLEMENTAL METHODS 2](#_Toc215657507)

[Search Strategy 2](#_Toc215657508)

[SUPPLEMENTAL MATERIAL 5](#_Toc215657509)

[Supplemental Figure S1 5](#_Toc215657510)

[Supplemental Figure S2 9](#_Toc215657511)

[SUPPLEMENTAL TABLES 13](#_Toc215657512)

[Supplemental Table S1 13](#_Toc215657513)

[Supplemental Table S2 16](#_Toc215657514)

[Supplemental Table S3 17](#_Toc215657515)

[Supplemental Table S4 20](#_Toc215657516)

[Supplemental Table S5 22](#_Toc215657517)

[Supplemental Table S6 22](#_Toc215657518)

# SUPPLEMENTAL METHODS

## Search Strategy

Overall search strategy:

Free text search strategy: (("sleep apnea" OR "sleep apnoea" OR "nocturnal hypoxia" OR "nocturnal hypoxaemia" OR "nocturnal hypoxemia" OR "sleep disordered breathing") AND ("artificial intelligence" OR "machine learning" OR "deep learning" OR "logistic regression" OR "support vector machine" OR "neural network" OR "classification tree" OR "regression tree" or "probability tree" OR "nearest neighbour" OR "nearest neighbor" OR "fuzzy logic" OR "naive bayes" OR "genetic algorithm" OR "multilayer perceptron" OR "random forest" OR "lasso regression" OR "kernel regression" OR “elastic net" OR "generative model" OR "generative adversarial network" OR "large language model") AND (diagnosis OR diagnose OR detect OR detection OR identify OR identification OR severity OR classify OR classification))

Pubmed search strategy:

("sleep apnea"[All Fields] OR "sleep apnoea"[All Fields] OR "nocturnal hypoxia"[All Fields] OR "nocturnal hypoxaemia"[All Fields] OR "nocturnal hypoxemia"[All Fields] OR "sleep disordered breathing"[All Fields]) AND ("artificial intelligence"[All Fields] OR "machine learning"[All Fields] OR "deep learning"[All Fields] OR "logistic regression"[All Fields] OR "support vector machine"[All Fields] OR "neural network"[All Fields] OR "classification tree"[All Fields] OR "regression tree"[All Fields] OR "probability tree"[All Fields] OR "nearest neighbour"[All Fields] OR "nearest neighbor"[All Fields] OR "fuzzy logic"[All Fields] OR "naive bayes"[All Fields] OR "genetic algorithm"[All Fields] OR "multilayer perceptron"[All Fields] OR "random forest"[All Fields] OR "lasso regression"[All Fields] OR "kernel regression"[All Fields] OR "elastic net"[All Fields] OR "generative model"[All Fields] OR "generative adversarial network"[All Fields] OR "large language model"[All Fields]) AND ("diagnosable"[All Fields] OR "diagnosi"[All Fields] OR "diagnosis"[MeSH Terms] OR "diagnosis"[All Fields] OR "diagnose"[All Fields] OR "diagnosed"[All Fields] OR "diagnoses"[All Fields] OR "diagnosing"[All Fields] OR "diagnosis"[MeSH Subheading] OR ("diagnosable"[All Fields] OR "diagnosi"[All Fields] OR "diagnosis"[MeSH Terms] OR "diagnosis"[All Fields] OR "diagnose"[All Fields] OR "diagnosed"[All Fields] OR "diagnoses"[All Fields] OR "diagnosing"[All Fields] OR "diagnosis"[MeSH Subheading]) OR ("detect"[All Fields] OR "detectabilities"[All Fields] OR "detectability"[All Fields] OR "detectable"[All Fields] OR "detectables"[All Fields] OR "detectably"[All Fields] OR "detected"[All Fields] OR "detectible"[All Fields] OR "detecting"[All Fields] OR "detection"[All Fields] OR "detections"[All Fields] OR "detects"[All Fields]) OR ("detect"[All Fields] OR "detectabilities"[All Fields] OR "detectability"[All Fields] OR "detectable"[All Fields] OR "detectables"[All Fields] OR "detectably"[All Fields] OR "detected"[All Fields] OR "detectible"[All Fields] OR "detecting"[All Fields] OR "detection"[All Fields] OR "detections"[All Fields] OR "detects"[All Fields]) OR ("identifiable"[All Fields] OR "identifiably"[All Fields] OR "identifie"[All Fields] OR "identified"[All Fields] OR "identifier"[All Fields] OR "identifiers"[All Fields] OR "identifies"[All Fields] OR "identify"[All Fields] OR "identifying"[All Fields]) OR ("identifed"[All Fields] OR "identification, psychological"[MeSH Terms] OR ("identification"[All Fields] AND "psychological"[All Fields]) OR "psychological identification"[All Fields] OR "identification"[All Fields] OR "identifications"[All Fields]) OR ("sever"[All Fields] OR "severe"[All Fields] OR "severed"[All Fields] OR "severely"[All Fields] OR "severer"[All Fields] OR "severes"[All Fields] OR "severing"[All Fields] OR "severities"[All Fields] OR "severity"[All Fields] OR "severs"[All Fields]) OR ("classifiable"[All Fields] OR "classification"[MeSH Terms] OR "classification"[All Fields] OR "classified"[All Fields] OR "classify"[All Fields] OR "classifying"[All Fields] OR "classifier"[All Fields] OR "classifier s"[All Fields] OR "classifiers"[All Fields] OR "classifies"[All Fields]) OR ("classification"[MeSH Terms] OR "classification"[All Fields] OR "classifications"[All Fields] OR "classification"[MeSH Subheading] OR "classification s"[All Fields] OR "classificator"[All Fields] OR "classificators"[All Fields]))

Embase search strategy:

('sleep apnea'/exp OR 'sleep apnea' OR 'sleep apnoea'/exp OR 'sleep apnoea' OR 'nocturnal hypoxia'/exp OR 'nocturnal hypoxia' OR 'nocturnal hypoxaemia' OR 'nocturnal hypoxemia'/exp OR 'nocturnal hypoxemia' OR 'sleep disordered breathing'/exp OR 'sleep disordered breathing') AND ('artificial intelligence'/exp OR 'artificial intelligence' OR 'machine learning'/exp OR 'machine learning' OR 'deep learning'/exp OR 'deep learning' OR 'logistic regression'/exp OR 'logistic regression' OR 'support vector machine'/exp OR 'support vector machine' OR 'neural network'/exp OR 'neural network' OR 'classification tree'/exp OR 'classification tree' OR 'regression tree'/exp OR 'regression tree' OR 'probability tree' OR 'nearest neighbour' OR 'nearest neighbor' OR 'fuzzy logic'/exp OR 'fuzzy logic' OR 'naive bayes'/exp OR 'naive bayes' OR 'genetic algorithm'/exp OR 'genetic algorithm' OR 'multilayer perceptron'/exp OR 'multilayer perceptron' OR 'random forest'/exp OR 'random forest' OR 'lasso regression'/exp OR 'lasso regression' OR 'kernel regression'/exp OR 'kernel regression' OR 'elastic net'/exp OR 'elastic net' OR 'generative model'/exp OR 'generative model' OR 'generative adversarial network'/exp OR 'generative adversarial network' OR 'large language model') AND ('diagnosis'/exp OR diagnosis OR diagnose OR detect OR 'detection'/exp OR detection OR identify OR 'identification'/exp OR identification OR 'severity'/exp OR severity OR classify OR 'classification'/exp OR classification) NOT [medline]/lim AND ([article]/lim OR [article in press]/lim) AND [english]/lim

Scopus search strategy:

( "sleep apnea" OR "sleep apnoea" OR "nocturnal hypoxia" OR "nocturnal hypoxaemia" OR "nocturnal hypoxemia" OR "sleep disordered breathing" ) AND ( "artificial intelligence" OR "machine learning" OR "deep learning" OR "logistic regression" OR "support vector machine" OR "neural network" OR "classification tree" OR "regression tree" OR "probability tree" OR "nearest neighbour" OR "nearest neighbor" OR "fuzzy logic" OR "naive bayes" OR "genetic algorithm" OR "multilayer perceptron" OR "random forest" OR "lasso regression" OR "kernel regression" OR "elastic net" OR "generative model" OR "generative adversarial network" OR "large language model" ) AND ( diagnosis OR diagnose OR detect OR detection OR identify OR identification OR severity OR classify OR classification ) AND NOT INDEX ( medline ) AND ( LIMIT-TO ( DOCTYPE , "ar" ) ) AND ( LIMIT-TO ( LANGUAGE , "English" ) ) AND ( LIMIT-TO ( EXACTKEYWORD , "Human" ) )

Scopus search strategy:

( "sleep apnea" OR "sleep apnoea" OR "nocturnal hypoxia" OR "nocturnal hypoxaemia" OR "nocturnal hypoxemia" OR "sleep disordered breathing" ) AND ( "artificial intelligence" OR "machine learning" OR "deep learning" OR "logistic regression" OR "support vector machine" OR "neural network" OR "classification tree" OR "regression tree" OR "probability tree" OR "nearest neighbour" OR "nearest neighbor" OR "fuzzy logic" OR "naive bayes" OR "genetic algorithm" OR "multilayer perceptron" OR "random forest" OR "lasso regression" OR "kernel regression" OR "elastic net" OR "generative model" OR "generative adversarial network" OR "large language model" ) AND ( diagnosis OR diagnose OR detect OR detection OR identify OR identification OR severity OR classify OR classification ) AND NOT INDEX ( medline ) AND ( LIMIT-TO ( DOCTYPE , "ar" ) ) AND ( LIMIT-TO ( LANGUAGE , "English" ) )

Web of science search strategy:

("sleep apnea" OR "sleep apnoea" OR "nocturnal hypoxia" OR "nocturnal hypoxaemia" OR "nocturnal hypoxemia" OR "sleep disordered breathing") AND ("artificial intelligence" OR "machine learning" OR "deep learning" OR "logistic regression" OR "support vector machine" OR "neural network" OR "classification tree" OR "regression tree" or "probability tree" OR "nearest neighbour" OR "nearest neighbor" OR "fuzzy logic" OR "naive bayes" OR "genetic algorithm" OR "multilayer perceptron" OR "random forest" OR "lasso regression" OR "kernel regression" OR “elastic net" OR "generative model" OR "generative adversarial network" OR "large language model") AND (diagnosis OR diagnose OR detect OR detection OR identify OR identification OR severity OR classify OR classification)

With the following limits applied: article, English language

IEEE Xplore search strategy:

("sleep apnea" OR "nocturnal hypoxemia" OR "sleep disordered breathing") AND ("artificial intelligence" OR "machine learning" OR "deep learning" OR "logistic regression" OR "support vector machine" OR "neural network" OR "classification tree" OR "regression tree" or "probability tree" OR "nearest neighbor" OR "random forest" OR "generative model" OR "generative adversarial network" OR "large language model") AND (diagnos* OR detect* OR identif* OR severity OR classif*)

# SUPPLEMENTAL MATERIAL

Supplemental Figure S1**: Accuracy versus covariate plot for Bayesian meta-regression of (A) classifier (Decision Trees [DT], Gradient Boosting [GB], Linear Model [LM], Neural Network [NN], Support Vector Machine [SVM]), (B) feature engineering (deep learning/domain expert), (C) model evaluation (random split/cross-validation), (D) AHI diagnostic cut offs (5, 10, 15, 30)**

**Supplemental Figure S1A:**


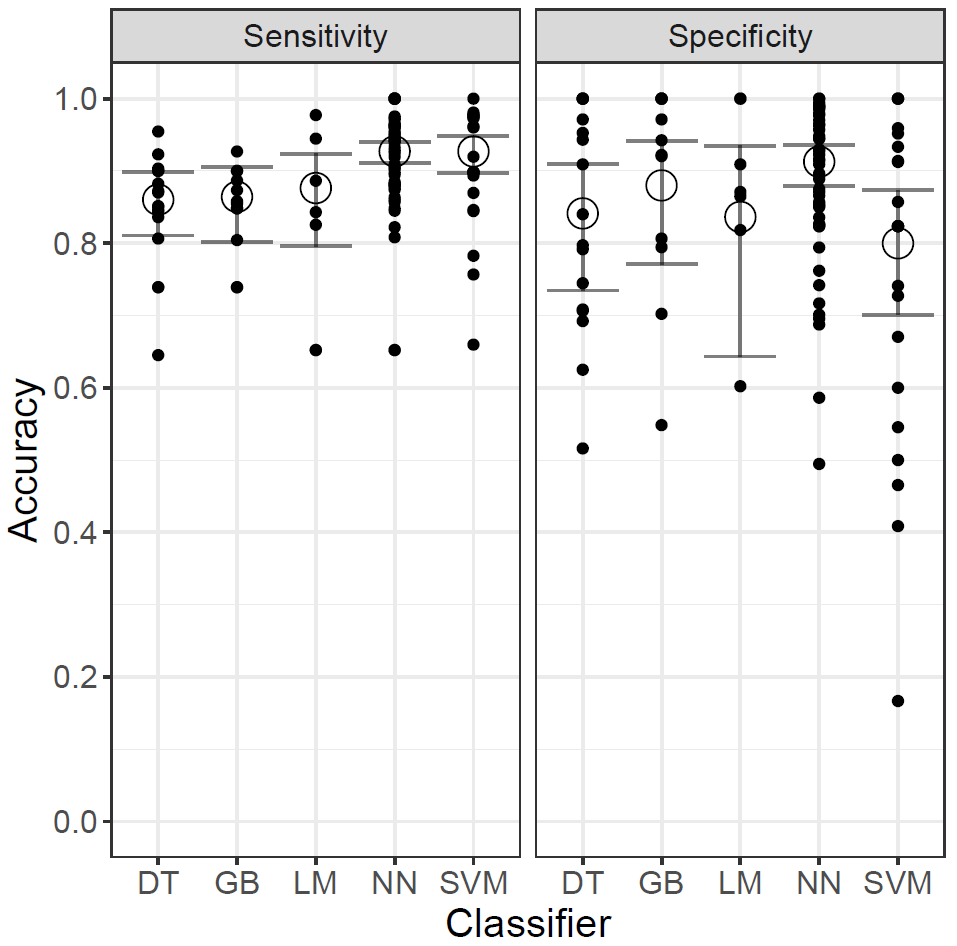


**Supplemental Figure S1B:**


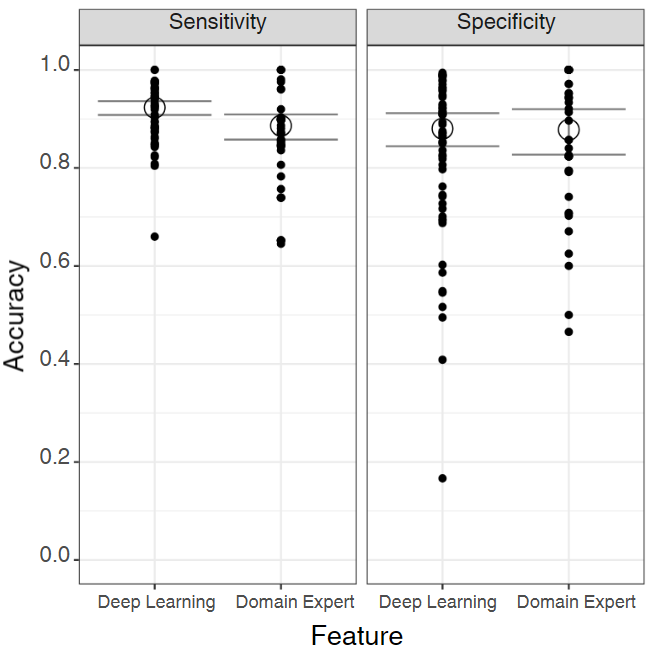


**Supplemental Figure S1C:**


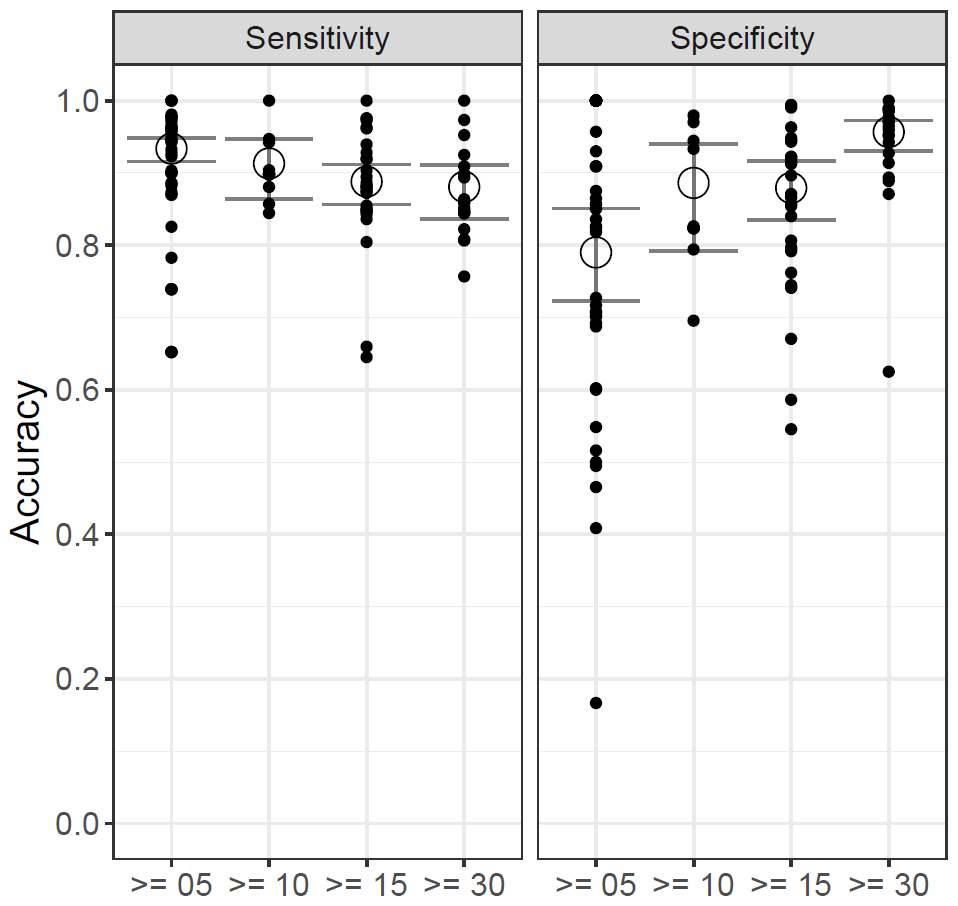


**Supplemental Figure S1D:**


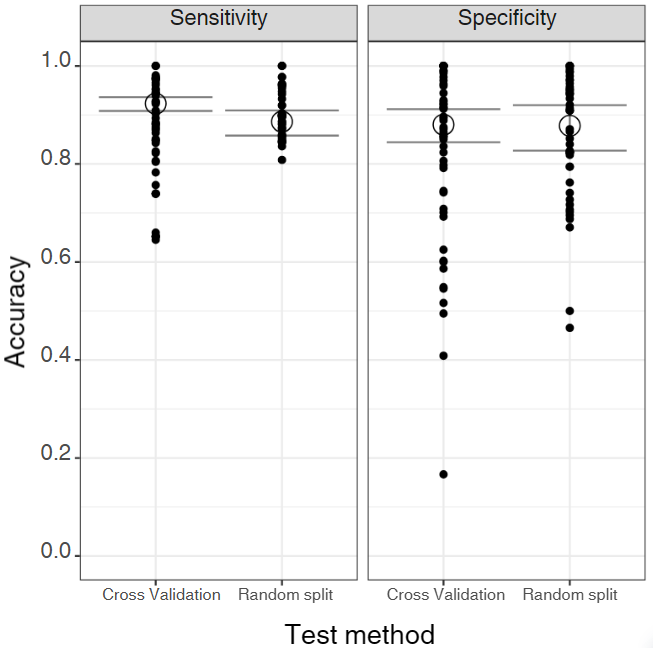


Supplemental Figure S2**: Accuracy versus covariate plot for Bayesian meta-regression of (A) percentage prevalence, (B) average age, (C) percentage male**

**Supplemental Figure S2A:**


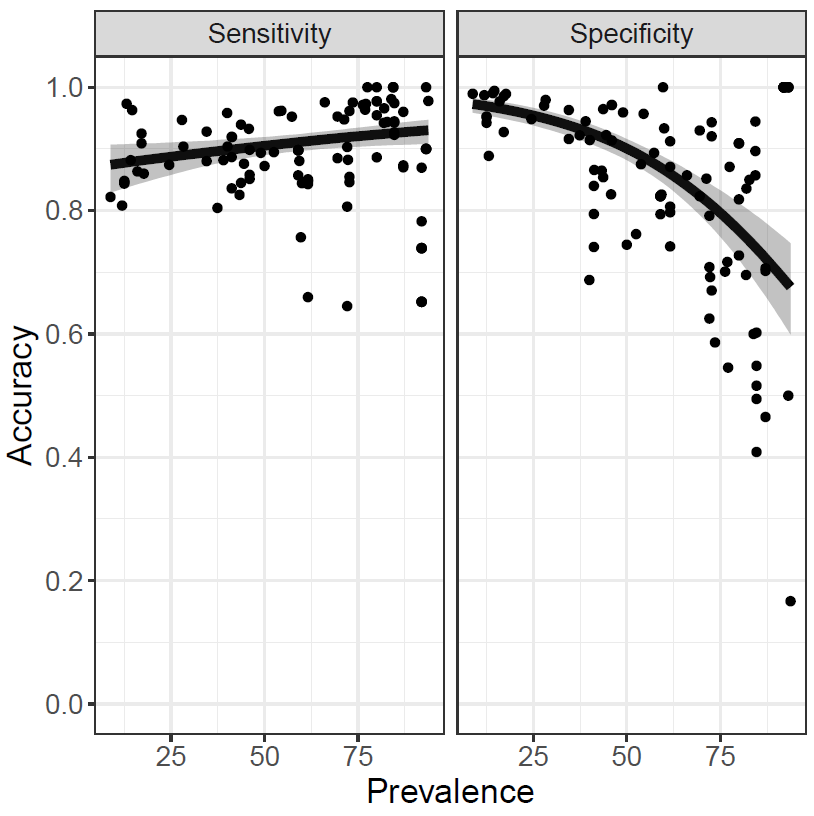


**Supplemental Figure S2B:**


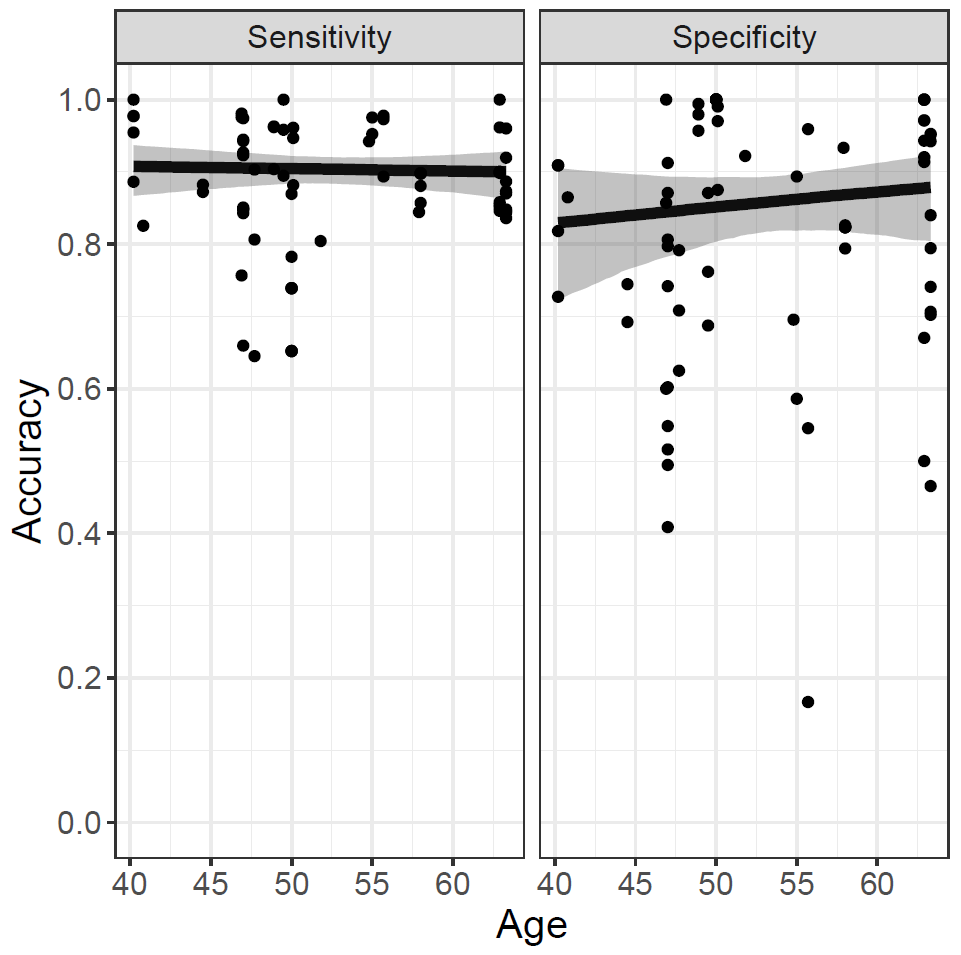


**Supplemental Figure S2C:**


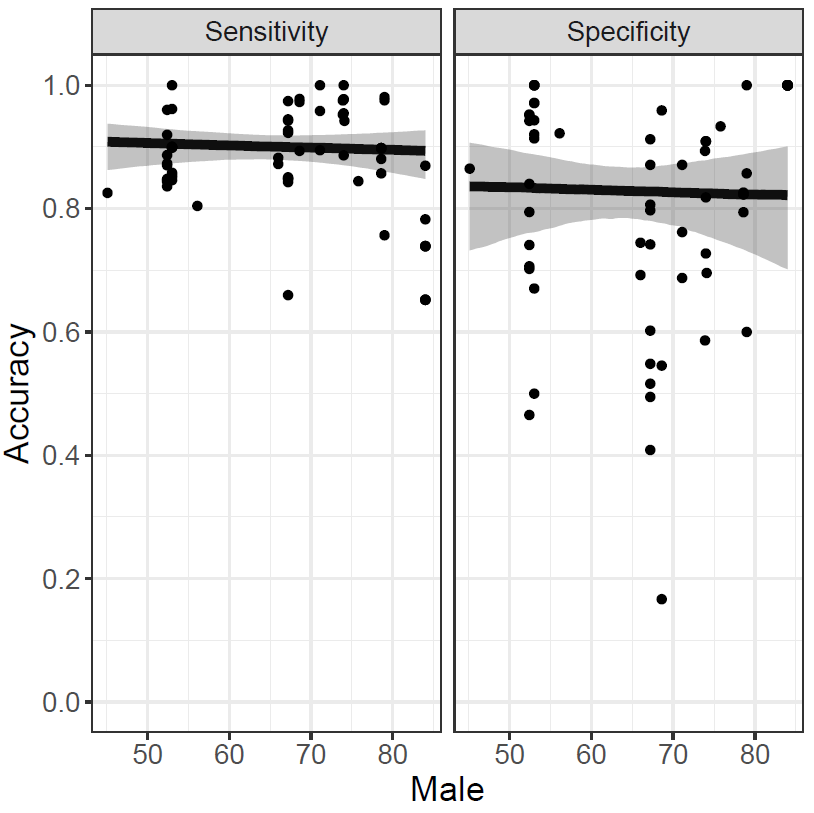


Supplemental Figure S3**: Accuracy versus covariate plot for Bayesian meta-regression of (A) Sampling frequency, (B) Sampling frequency**

**Supplemental Figure S3A:**


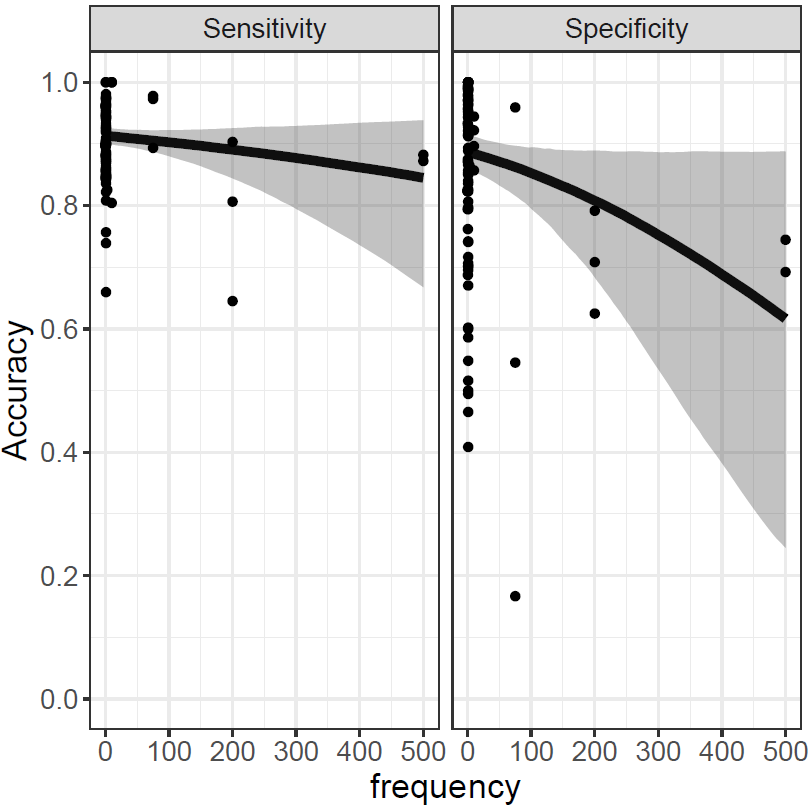


**Supplemental Figure S3B:**


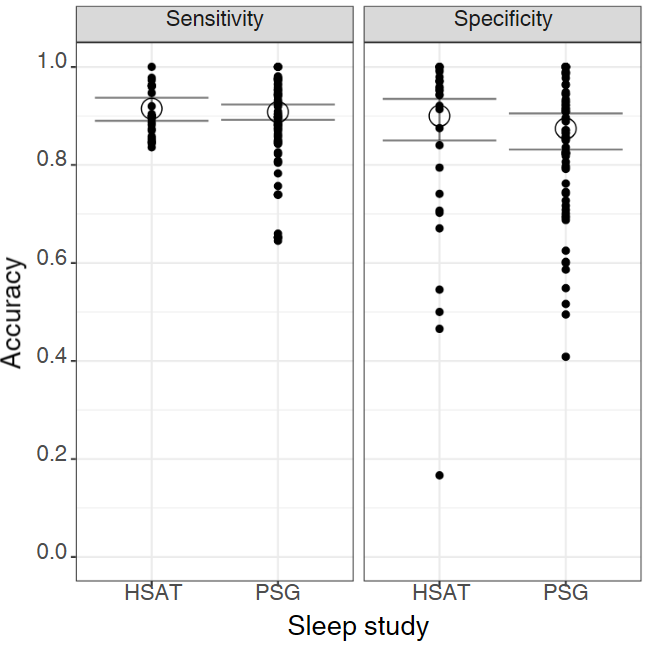


# SUPPLEMENTAL TABLES

Supplemental Table S1**: Summary of included studies.**

| **First Author & Year DOI** | **Reference Standard** | **OSA Prevalence for each AHI** | **Country** | **Sample Size Average Age % Male** | **Feature Engineering Classification Model Evaluation** |
| --- | --- | --- | --- | --- | --- |
| Alvarez et al (2020) [37] | HSAT AHI >=5, 15, 30 | 93.8, 77.1, 49 | Europe | 96 55.7 68.6 | Deep Learning SVM Cross-validation |
| Alvarez et al (2016) [38] | PSG AHI >=10 | 81.9 | Europe | 127 54.8 74.1 | Deep Learning NN Random split |
| Andres-Blanco et al (2017) [12] | PSG AHI >=15, 30 | 73.6, 57.3 | Europe | 110 55 73.9 | Deep Learning NN Cross-validation |
| Behar et al (2019) [13] | PSG AHI >=5 | 43.3 | South America | 887 40.8 45.1 | Deep Learning LR  Cross-validation |
| Cajal et al (2023) [39] | PSG AHI >=5, 15 | 72.3, 50 | Europe | 94 44.5 66 | Deep Learning CART Cross-validation |
| Chen et al (2023) [20] | PSG AHI >=5, 15, 30 | 69.5, 34.5, 13.1 | North America | 5793 NR NR | Deep Learning  NN  Cross-validation |
| Gutierrez-Tobal et al  (2021) [40] | HSAT AHI >=5, 15, 30 | 87.1, 41.2, 12.5 (SHHS1)  93.2, 72.7, 46 (RHUH) | North America | 3689 (SHHS1), 322 (RHUH) 63.3 (SHHS1), 62.9 (RHUH) 52.4 (SHHS1), 53 (RHUH) | Domain Expert  GB, SVM, CART  Random split |
| Kaimakamis et al  (2009) [41] | PSG AHI >=5, 15, 30 | 72.1, 72.1, 72.1 | Europe | 86 47.7 NR | Domain Expert CART Cross-validation |
| Kuo et al (2025) [58] | PSG AHI >=15 | 37.4 | Asia | 123  51.8  56.1 | Deep Learning  GB  Cross-validation |
| Leong et al (2023) [42] | PSG AHI >=5, 15 | 84.7, 61.6 | Asia | 599 47 67.2 | Deep Learning LR, RF, SVM, GB, NN Cross-validation |
| Li et al (2021) [43] | PSG AHI >=5 | 80 | Asia | 55 40.2 74 | Deep Learning  LDA, SVM, CART, LR, NN  Random split |
| Li et al (2025) [57] | PSG AHI >=5, 15, 30 | 82.7, 44.5, 17.1 (SHHS1)  82, 43.7, 17.1 (SHHS2) | North America | 580 (SHHS1), 2643 (SHHS2)  NR  NR | Deep Learning  NN  Cross-validation |
| Ma et al (2019) [44] | PSG AHI >=5 | 92 | Europe | 25 50 84 | Domain expert SVM, GB Cross-validation |
| Marcos et al (2007) [45] | PSG AHI >=10 | 59 | Europe | 83 58 78.6 | Domain expert NN, SVM Random split |
| Marcos et al (2008) [46] | PSG AHI >=10 | 59 | Europe | 83 58 78.6 | Domain expert SVM Random split |
| Marcos et al (2008) [47] | PSG AHI >=10 | 59 | Europe | 83 58 78.6 | Domain expert NN  Random split |
| Marcos et al (2009) [48] | PSG AHI >=10 | 60 | Europe | 75 57.9  75.8 | Deep Learning  SVM  Random split |
| Marcos et al (2010) [49] | PSG AHI >=10 | 59.3 | Europe | 113 58 78.6 | Domain expert NN  Random split |
| Muthukumaran et al (2025) [56] | PSG AHI >=5, 15, 30 | 71.3, 39, 17.7 (SHHS1)  76.9, 41.3, 17.2 (SHHS2)  45.8, 24.5, 11.9 (CFS) | North America | 564 (SHHS1), 750 (SHHS2), 616 (CFS)  NR  NR | Deep Learning  NN  Random Split |
| Nikkonen et al (2019) [50] | HSAT AHI >=5, 10, 15 | 54.5, 28.3, 14.6 (Random split)  53.8, 27.9, 14.2 (Cross-validation) | Europe | 198 (Random split), 1959 (Cross-validation)  48.9 (Random split), 50.1 (Cross-validation) NR | Deep Learning  NN  Random split, Cross-validation |
| Peng et al (2024) [51] | PSG AHI >=5, 10, 15 | 84.4, 84.4, 84.4 | Asia | 45  NR  NR | Domain Expert  NN  Cross-validation |
| Polat et al (2008) [52] | PSG AHI >=5, 15, 30 | 40, 52.5, 77.5 | Asia | 40  49.5  71.1 | Deep Learning  NN  Random split |
| Wu et al (2018) [53] | PSG AHI >=5, 15, 30 | 83.9, 66.1, 59.7 | Asia | 62  46.9  79 | Domain Expert  SVM  Cross-validation |
| Wu et al (2025) [55] | PSG AHI >=5, 15, 30 | 69.5, 34.5, 8.8 (SHHS1)  76.3, 40.1, 16 (SHHS2) | North America | 5793 (SHHS1), 2651 (SHHS2)  NR  NR | Deep Learning  NN  Cross-validation |
| Zhang et al (2023) [54] | PSG AHI >=5 | 92 | Europe | 25  50  84 | Domain Expert  SVM, NN, RF, LDA  Cross-validation |

Abbreviations: PSG, polysomnography; HSAT, home sleep apnea test; AHI, apnea-hypopnea index; SVM, Support Vector Machine; NN, Neural Network; RF, Random Forest; LDA, Linear Discriminant Analysis; GB, Gradient Boosting; LR, Logistic Regression; CART, Classification and Regression Tree

Supplemental Table S2**: Evaluation of risk of bias using the Quality Assessment of Diagnostic Accuracy Studies-2 (QUADAS-2) tool for diagnostic accuracy.**

| **Study** | **QUADAS-2 risk of bias** | | | | **QUADAS-2 concerns regarding applicability** | | | **Risk of bias** |
| --- | --- | --- | --- | --- | --- | --- | --- | --- |
|  | **Patient Selection** | **Index Test** | **Reference Standard** | **Flow & Timing** | **Patient Selection** | **Index Test** | **Reference Standard** |  |
| Alvarez et al (2020) [37] | Low | Low | Low | Low | Low | Low | Low | Low |
| Alvarez et al (2016) [38] | Unclear | Low | Low | Low | Low | Unclear | Low | Unclear |
| Andres-Blanco et al (2017) [12] | Low | Low | Low | Low | Low | Unclear | Low | Unclear |
| Behar et al (2019) [13] | Unclear | Low | Low | Low | Low | Unclear | Low | Unclear |
| Cajal et al (2023) [39] | Unclear | Low | Low | Low | Low | Low | Low | Low |
| Chen et al (2023) [20] | Low | Low | Low | Unclear | Low | Low | Low | Unclear |
| Gutierrez-Tobal et al  (2021) [40] | Low | Low | Low | Unclear | Low | Low | Low | Unclear |
| Kaimakamis et al  (2009) [41] | Low | Low | Low | Low | Low | Low | Low | Low |
| Kuo et al (2025) [58] | Low | Unclear | Unclear | Low | Low | Low | Low | Unclear |
| Leong et al (2023) [42] | Low | Low | Low | Unclear | Low | Low | Low | Unclear |
| Li et al (2021) [43] | Low | Low | Low | Low | Low | Low | Low | Low |
| Li et al (2025) [57] | Low | Unclear | Low | Low | Low | Low | Low | Unclear |
| Ma et al (2019) [44] | Unclear | Low | Low | Low | Unclear | Low | Low | Unclear |
| Marcos et al (2007) [45] | Unclear | Low | Low | Low | Unclear | Low | Low | Unclear |
| Marcos et al (2008) [46] | Unclear | Low | Low | Low | Unclear | Low | Low | Unclear |
| Marcos et al (2008) [47] | Unclear | Low | Low | Low | Unclear | Low | Low | Unclear |
| Marcos et al (2009) [48] | Low | Unclear | Low | Low | Low | Unclear | Low | Unclear |
| Marcos et al (2010) [49] | Unclear | Low | Low | Low | Unclear | Low | Low | Unclear |
| Muthukumaran et al (2025) [56] | Low | Unclear | Unclear | Low | Low | Low | Low | Unclear |
| Nikkonen et al (2019) [50] | Low | Unclear | Low | Low | Low | Unclear | Low | Unclear |
| Peng et al (2024) [51] | Unclear | Unclear | Unclear | Low | Unclear | Unclear | Low | Unclear |
| Polat et al (2008) [52] | Unclear | Low | Low | Low | Unclear | Low | Low | Unclear |
| Wu et al (2018) [53] | Low | Unclear | Unclear | Low | Low | Unclear | Unclear | Unclear |
| Wu et al (2025) [55] | Low | Low | Low | Low | Low | Low | Low | Low |
| Zhang et al (2023) [54] | Low | Low | Low | Low | Low | Low | Low | Low |

Supplemental Table S3**: Pulse oximeter brand and Sampling frequency used by each study**

| Author | Oximeter brand | Sampling frequency (Hz) |
| --- | --- | --- |
| Alvarez et al (2020) [37] | Nonin XPOD | 75 |
| Alvarez et al (2016) [38] | Nonin WristOx2 3150 | 1 |
| Andres-Blanco et al (2017) [12] | Nonin WristOx2 3150 | 1 |
| Behar et al (2019) [13] | Nonin XPOD | 3 |
| Cajal et al (2023) [39] | Nonin 8000J | 500 |
| Chen et al (2023) [20] | Nonin (unspecified) | 1 |
| Gutierrez-Tobal et al  (2021) [40] | Nonin (unspecified) | 1 |
| Kaimakamis et al  (2009) [41] | Nonin XPOD | 200 |
| Kuo et al (2025) [58] | Embla N7000 | 1 |
| Leong et al (2023) [42] | Grael PSG (unspecified) | 1 |
| Li et al (2021) [43] | Unspecified | Unspecified |
| Li et al (2025) [57] | Nonin (unspecified) | 1 |
| Ma et al (2019) [44] | Unspecified | 1 |
| Marcos et al (2007) [45] | Criticare 504 oximeter | 0.2 |
| Marcos et al (2008) [46] | Criticare 504 oximeter | 0.2 |
| Marcos et al (2008) [47] | Criticare 504 oximeter | 0.2 |
| Marcos et al (2009) [48] | Criticare 504 oximeter | 0.2 |
| Marcos et al (2010) [49] | Criticare 504 oximeter | 0.2 |
| Muthukumaran et al (2025) [56] | Nonin XPOD 3011 | 1 |
| Nikkonen et al (2019) [50] | Nonin XPOD 3012 | 0.5 |
| Peng et al (2024) [51] | SleepSense Adult Soft-Tip SpO2 Sensor | 10 |
| Polat et al (2008) [52] | Unspecified | 0.5 |
| Wu et al (2018) [53] | Masimo Oximeter | 1 |
| Wu et al (2025) [55] | Nonin (unspecified) | 1 |
| Zhang et al (2023) [54] | Unspecified | Unspecified |

Supplemental Table S4A**: Summary Statistics for each study**

| Study | HSAT or PSG / AHI cut-offs | AI Classifier | TP | FN | TN | FP | Accuracy | Sn | Sp | Calculation done? |
| --- | --- | --- | --- | --- | --- | --- | --- | --- | --- | --- |
| Alvarez et al (2020) [37] | HSAT AHI >= 5 | SVM | 88 | 2 | 1 | 5 | 92.13 | 97.23 | 33.70 | No |
| Alvarez et al (2020) [37] | HSAT AHI >= 15 | SVM | 72 | 2 | 12 | 10 | 90.17 | 95.33 | 72.07 | No |
| Alvarez et al (2020) [37] | HSAT AHI >= 30 | SVM | 42 | 5 | 47 | 2 | 87.07 | 96.57 | 54.33 | No |
| Alvarez et al (2016) [38] | PSG AHI >= 10 | NN | 98 | 6 | 16 | 7 | 90.17 | 95.33 | 72.07 | No |
| Andres-Blanco et al (2017) [12] | PSG AHI >= 15 | NN | 79 | 2 | 17 | 12 | 92.60 | 88.93 | 94.87 | Yes, from Sn and Sp |
| Andres-Blanco et al (2017) [12] | PSG AHI >= 30 | NN | 60 | 3 | 42 | 5 | 95.37 | 92.93 | 97.00 | Yes, from Sn and Sp |
| Behar et al (2019) [13] | PSG AHI >= 5 | LR | 317 | 67 | 435 | 68 | 89.80 | 94.20 | 69.60 | Yes, from Sn and Sp |
| Cajal et al (2023) [39] | PSG AHI >= 5 | CART | 60 | 8 | 18 | 8 | 87.30 | 97.50 | 58.60 | Yes, from Sn and Sp |
| Cajal et al (2023) [39] | PSG AHI >= 15 | CART | 41 | 6 | 35 | 12 | 92.70 | 95.20 | 89.40 | Yes, from Sn and Sp |
| Chen et al (2023) [20] | PSG AHI >= 5 | NN | 1477 | 192 | 3835 | 289 | 85.00 | 83.00 | 86.00 | No |
| Chen et al (2023) [20] | PSG AHI >= 15 | NN | 3640 | 282 | 1714 | 157 | 72.73 | 87.50 | 69.44 | No |
| Chen et al (2023) [20] | PSG AHI >= 30 | NN | 4956 | 136 | 623 | 78 | 79.55 | 88.24 | 74.07 | No |
| Gutierrez-Tobal et al  (2021) [40] | HSAT AHI >= 5 | GB | 2805 | 407 | 335 | 142 | 91.70 | 88.50 | 92.99 | No |
| Gutierrez-Tobal et al  (2021) [40] | HSAT AHI >= 5 | SVM | 3084 | 128 | 222 | 255 | 92.42 | 92.81 | 91.61 | No |
| Gutierrez-Tobal et al  (2021) [40] | HSAT AHI >= 5 | CART | 2795 | 417 | 337 | 140 | 96.31 | 97.33 | 88.87 | No |
| Gutierrez-Tobal et al  (2021) [40] | HSAT AHI >= 15 | GB | 1347 | 172 | 1724 | 446 | 85.12 | 87.32 | 70.23 | No |
| Gutierrez-Tobal et al  (2021) [40] | HSAT AHI >= 15 | SVM | 1397 | 122 | 1608 | 562 | 89.62 | 96.01 | 46.54 | No |
| Gutierrez-Tobal et al  (2021) [40] | HSAT AHI >= 15 | CART | 1270 | 249 | 1823 | 347 | 84.90 | 87.02 | 70.65 | No |
| Gutierrez-Tobal et al  (2021) [40] | HSAT AHI >= 30 | GB | 391 | 70 | 3042 | 186 | 83.25 | 88.67 | 79.44 | No |
| Gutierrez-Tobal et al  (2021) [40] | HSAT AHI >= 30 | SVM | 390 | 71 | 3072 | 156 | 81.46 | 91.97 | 74.10 | No |
| Gutierrez-Tobal et al  (2021) [40] | HSAT AHI >= 30 | CART | 389 | 72 | 3075 | 153 | 83.84 | 83.61 | 84.00 | No |
| Gutierrez-Tobal et al  (2021) [40] | HSAT AHI >= 5 | GB | 270 | 30 | 22 | 0 | 93.06 | 84.81 | 94.24 | No |
| Gutierrez-Tobal et al  (2021) [40] | HSAT AHI >= 5 | SVM | 300 | 0 | 11 | 11 | 93.85 | 84.60 | 95.17 | No |
| Gutierrez-Tobal et al  (2021) [40] | HSAT AHI >= 5 | CART | 270 | 30 | 22 | 0 | 93.90 | 84.38 | 95.26 | No |
| Gutierrez-Tobal et al  (2021) [40] | HSAT AHI >= 15 | GB | 200 | 34 | 81 | 7 | 90.68 | 90.00 | 100.00 | No |
| Gutierrez-Tobal et al  (2021) [40] | HSAT AHI >= 15 | SVM | 225 | 9 | 59 | 29 | 96.58 | 100.00 | 50.00 | No |
| Gutierrez-Tobal et al  (2021) [40] | HSAT AHI >= 15 | CART | 198 | 36 | 83 | 5 | 90.68 | 90.00 | 100.00 | No |
| Gutierrez-Tobal et al  (2021) [40] | HSAT AHI >= 30 | GB | 127 | 21 | 169 | 5 | 87.27 | 85.47 | 92.05 | No |
| Gutierrez-Tobal et al  (2021) [40] | HSAT AHI >= 30 | SVM | 133 | 15 | 159 | 15 | 88.20 | 96.15 | 67.04 | No |
| Gutierrez-Tobal et al  (2021) [40] | HSAT AHI >= 30 | CART | 126 | 22 | 169 | 5 | 87.27 | 84.62 | 94.32 | No |
| Kaimakamis et al  (2009) [41] | PSG AHI >= 5 | CART | 56 | 6 | 17 | 7 | 91.93 | 85.81 | 97.13 | No |
| Kaimakamis et al  (2009) [41] | PSG AHI >= 15 | CART | 40 | 22 | 19 | 5 | 90.68 | 89.86 | 91.38 | No |
| Kaimakamis et al  (2009) [41] | PSG AHI >= 30 | CART | 50 | 12 | 15 | 9 | 91.61 | 85.14 | 97.13 | No |
| Kuo et al (2025) [58] | PSG AHI >= 15 | GB | 66 | 16 | 29 | 12 | 37.4 | 80.00 | 77.32 | Yes, from accuracy and recall |
| Leong et al (2023) [42] | PSG AHI >= 5 | LR | 478 | 28 | 56 | 37 | 89.10 | 94.50 | 60.60 | No |
| Leong et al (2023) [42] | PSG AHI >= 5 | RF | 467 | 39 | 48 | 45 | 86.00 | 92.30 | 52.10 | No |
| Leong et al (2023) [42] | PSG AHI >= 5 | SVM | 493 | 13 | 38 | 55 | 88.50 | 97.40 | 40.40 | No |
| Leong et al (2023) [42] | PSG AHI >= 5 | GB | 469 | 37 | 51 | 42 | 86.60 | 92.70 | 54.30 | No |
| Leong et al (2023) [42] | PSG AHI >= 5 | NN | 477 | 29 | 46 | 47 | 90.20 | 95.60 | 60.60 | No |
| Leong et al (2023) [42] | PSG AHI >= 15 | LR | 322 | 60 | 189 | 28 | 85.30 | 84.30 | 87.10 | No |
| Leong et al (2023) [42] | PSG AHI >= 15 | RF | 325 | 57 | 173 | 44 | 83.10 | 85.10 | 79.70 | No |
| Leong et al (2023) [42] | PSG AHI >= 15 | SVM | 252 | 130 | 198 | 19 | 75.10 | 66.00 | 91.20 | No |
| Leong et al (2023) [42] | PSG AHI >= 15 | GB | 324 | 58 | 175 | 42 | 83.30 | 84.80 | 80.60 | No |
| Leong et al (2023) [42] | PSG AHI >= 15 | NN | 324 | 58 | 161 | 56 | 80.10 | 84.80 | 74.20 | No |
| Li et al (2021) [43] | PSG AHI >= 5 | LDA | 39 | 5 | 10 | 1 | 88.40 | 89.00 | 88.00 | Yes, from Sn and Sp |
| Li et al (2021) [43] | PSG AHI >= 5 | SVM | 43 | 1 | 8 | 3 | 93.40 | 97.00 | 76.00 | Yes, from Sn and Sp |
| Li et al (2021) [43] | PSG AHI >= 5 | CART | 42 | 2 | 10 | 1 | 93.90 | 95.00 | 91.00 | Yes, from Sn and Sp |
| Li et al (2021) [43] | PSG AHI >= 5 | LR | 43 | 1 | 9 | 2 | 95.00 | 98.00 | 82.00 | Yes, from Sn and Sp |
| Li et al (2021) [43] | PSG AHI >= 5 | NN | 44 | 0 | 10 | 1 | 98.10 | 100.00 | 90.90 | Yes, from Sn and Sp |
| Li et al (2025) [57] | PSG AHI >= 5 | NN | 453 | 27 | 85 | 15 | 92.76 | 94.38 | 85.00 | No |
| Li et al (2025) [57] | PSG AHI >= 15 | NN | 226 | 32 | 297 | 25 | 90.17 | 87.60 | 92.24 | No |
| Li et al (2025) [57] | PSG AHI >= 30 | NN | 90 | 9 | 474 | 7 | 97.24 | 90.91 | 98.54 | No |
| Li et al (2025) [57] | PSG AHI >= 5 | NN | 2094 | 74 | 397 | 78 | 94.25 | 96.59 | 83.58 | No |
| Li et al (2025) [57] | PSG AHI >= 15 | NN | 1086 | 70 | 1270 | 217 | 89.14 | 93.94 | 85.41 | No |
| Li et al (2025) [57] | PSG AHI >= 30 | NN | 419 | 34 | 2031 | 159 | 92.70 | 92.49 | 92.74 | No |
| Ma et al (2019) [44] | PSG AHI >= 5 | SVM | 20 | 3 | 2 | 0 | 90.20 | 87.60 | 94.10 | Yes, from Sn and Sp |
| Ma et al (2019) [44] | PSG AHI >= 5 | GB | 17 | 6 | 2 | 0 | 83.64 | 72.64 | 87.18 | Yes, from Sn and Sp |
| Marcos et al (2007) [45] | PSG AHI>= 10 | NN | 42 | 7 | 28 | 6 | 84.40 | 86.50 | 81.40 | Yes, from Sn and Sp |
| Marcos et al (2007) [45] | PSG AHI>= 10 | SVM | 44 | 5 | 28 | 6 | 86.30 | 89.90 | 81.10 | Yes, from Sn and Sp |
| Marcos et al (2008) [46] | PSG AHI>= 10 | SVM | 44 | 5 | 28 | 6 | 88.00 | 84.44 | 93.33 | Yes, from Sn and Sp |
| Marcos et al (2008) [47] | PSG AHI>= 10 | NN | 44 | 5 | 27 | 7 | 86.10 | 89.40 | 81.40 | Yes, from Sn and Sp |
| Marcos et al (2009) [48] | PSG AHI>= 10 | SVM | 38 | 7 | 28 | 2 | 85.50 | 89.80 | 79.40 | Yes, from Sn and Sp |
| Marcos et al (2010) [49] | PSG AHI>= 10 | NN | 59 | 8 | 38 | 8 | 85.58 | 87.76 | 82.39 | Yes, from Sn and Sp |
| Muthukumaran et al (2025) [56] | PSG AHI >= 5 | NN | 381 | 21 | 138 | 24 | 92.02 | 94.78 | 85.19 | No |
| Muthukumaran et al (2025) [56] | PSG AHI >= 15 | NN | 194 | 26 | 325 | 19 | 92.02 | 88.18 | 94.48 | No |
| Muthukumaran et al (2025) [56] | PSG AHI >= 30 | NN | 86 | 14 | 459 | 5 | 96.63 | 86.00 | 98.92 | No |
| Muthukumaran et al (2025) [56] | PSG AHI >= 5 | NN | 556 | 21 | 124 | 49 | 90.67 | 96.36 | 71.68 | No |
| Muthukumaran et al (2025) [56] | PSG AHI >= 15 | NN | 285 | 25 | 381 | 59 | 88.80 | 91.94 | 86.59 | No |
| Muthukumaran et al (2025) [56] | PSG AHI >= 30 | NN | 109 | 20 | 599 | 22 | 94.40 | 84.50 | 96.46 | No |
| Muthukumaran et al (2025) [56] | PSG AHI >= 5 | NN | 263 | 19 | 276 | 58 | 87.50 | 93.26 | 82.63 | No |
| Muthukumaran et al (2025) [56] | PSG AHI >= 15 | NN | 132 | 19 | 441 | 24 | 93.02 | 87.42 | 94.84 | No |
| Muthukumaran et al (2025) [56] | PSG AHI >= 30 | NN | 59 | 14 | 536 | 7 | 96.59 | 80.82 | 98.71 | No |
| Nikkonen et al (2019) [50] | HSAT AHI >= 5 | NN | 101 | 4 | 89 | 4 | 95.96 | 96.19 | 95.70 | No |
| Nikkonen et al (2019) [50] | HSAT AHI >= 10 | NN | 47 | 5 | 143 | 3 | 95.96 | 90.38 | 97.95 | No |
| Nikkonen et al (2019) [50] | HSAT AHI >= 15 | NN | 26 | 1 | 170 | 1 | 98.99 | 96.30 | 99.42 | No |
| Nikkonen et al (2019) [50] | HSAT AHI >= 5 | NN | 1013 | 41 | 792 | 113 | 92.14 | 96.11 | 87.51 | No |
| Nikkonen et al (2019) [50] | HSAT AHI >= 10 | NN | 518 | 29 | 1370 | 42 | 96.38 | 94.70 | 97.03 | No |
| Nikkonen et al (2019) [50] | HSAT AHI >= 15 | NN | 246 | 33 | 1664 | 16 | 97.50 | 88.17 | 99.05 | No |
| Peng et al (2024) [51] | PSG AHI >= 5 | NN | 38 | 0 | 6 | 1 | 97.78 | 100.00 | 85.71 | No |
| Peng et al (2024) [51] | PSG AHI >= 10 | NN | 27 | 0 | 17 | 1 | 97.78 | 100.00 | 94.44 | No |
| Peng et al (2024) [51] | PSG AHI >= 15 | NN | 16 | 0 | 26 | 3 | 93.33 | 100.00 | 89.66 | No |
| Polat et al (2008) [52] | PSG AHI >= 5 | NN | 23 | 1 | 11 | 5 | 85.00 | 95.83 | 68.75 | No |
| Polat et al (2008) [52] | PSG AHI >= 15 | NN | 17 | 2 | 16 | 5 | 82.50 | 89.47 | 76.19 | No |
| Polat et al (2008) [52] | PSG AHI >= 30 | NN | 9 | 0 | 27 | 4 | 90.00 | 100.00 | 87.10 | No |
| Wu et al (2018) [53] | PSG AHI >= 5 | SVM | 51 | 1 | 6 | 4 | 91.94 | 98.08 | 60.00 | No |
| Wu et al (2018) [53] | PSG AHI >= 15 | SVM | 40 | 1 | 18 | 3 | 93.55 | 97.56 | 85.71 | No |
| Wu et al (2018) [53] | PSG AHI >= 30 | SVM | 28 | 9 | 25 | 0 | 85.48 | 75.68 | 100.00 | No |
| Wu et al (2025) [55] | PSG AHI >= 5 | NN | 3836 | 191 | 1454 | 312 | 91.32 | 95.26 | 82.33 | No |
| Wu et al (2025) [55] | PSG AHI >= 15 | NN | 1757 | 239 | 3657 | 140 | 93.46 | 88.03 | 96.31 | No |
| Wu et al (2025) [55] | PSG AHI >= 30 | NN | 624 | 135 | 4981 | 53 | 96.75 | 82.21 | 98.95 | No |
| Wu et al (2025) [55] | PSG AHI >= 5 | NN | 1964 | 58 | 441 | 188 | 90.72 | 97.13 | 70.11 | No |
| Wu et al (2025) [55] | PSG AHI >= 15 | NN | 960 | 102 | 1453 | 136 | 91.02 | 90.40 | 91.44 | No |
| Wu et al (2025) [55] | PSG AHI >= 30 | NN | 367 | 58 | 2175 | 51 | 95.89 | 86.35 | 97.71 | No |
| Zhang et al (2023) [54] | PSG AHI >= 5 | SVM | 18 | 5 | 2 | 0 | 84.48 | 76.62 | 90.53 | Yes, from Sn and Sp |
| Zhang et al (2023) [54] | PSG AHI >= 5 | NN | 15 | 8 | 2 | 0 | 78.65 | 66.90 | 88.40 | Yes, from Sn and Sp |
| Zhang et al (2023) [54] | PSG AHI >= 5 | RF | 17 | 6 | 2 | 0 | 86.92 | 72.85 | 90.70 | Yes, from Sn and Sp |
| Zhang et al (2023) [54] | PSG AHI >= 5 | LDA | 15 | 8 | 2 | 0 | 80.60 | 63.52 | 87.55 | Yes, from Sn and Sp |

Abbreviations: PSG, polysomnography; HSAT, home sleep apnea test; AHI, apnea-hypopnea index; SVM, Support Vector Machine; NN, Neural Network; RF, Random Forest; LDA, Linear Discriminant Analysis; GB, Gradient Boosting; LR, Logistic Regression; CART, Classification and Regression Tree; Sn, Sensitivity; Sp, Specificity;

Supplemental Table S4B**: Datasets for each study**

| Study | HSAT or PSG / AHI cut-offs | AI Classifier | Dataset (Training) | | Dataset (Validation) | Dataset (Testing) |  |
| --- | --- | --- | --- | --- | --- | --- | --- |
| Alvarez et al (2020) [37] | HSAT AHI >= 5 | SVM | RHUH at home PSG, Jul 2016-Sept 2017, 303 samples before exclusion | | | |  |
| Alvarez et al (2020) [37] | HSAT AHI >= 15 | SVM | RHUH at home PSG, Jul 2016-Sept 2017, 303 samples before exclusion | | | |  |
| Alvarez et al (2020) [37] | HSAT AHI >= 30 | SVM | RHUH at home PSG, Jul 2016-Sept 2017, 303 samples before exclusion | | | |  |
| Alvarez et al (2016) [38] | PSG AHI >= 10 | NN | RHUH in-lab PSG, 320 samples | | | |  |
| Andres-Blanco et al (2017) [12] | PSG AHI >= 15 | NN | RHUH in-lab and home PSG, with COPD patients, Jun 2013-Jan2015 | | | |  |
| Andres-Blanco et al (2017) [12] | PSG AHI >= 30 | NN | RHUH in-lab and home PSG, with COPD patients, Jun 2013-Jan2015 | | | |  |
| Behar et al (2019) [13] | PSG AHI >= 5 | LR | EPISONO in-lab PSG | | | |  |
| Cajal et al (2023) [39] | PSG AHI >= 5 | CART | University Hospitals Leuven (Belgium) in-lab PSG | | | |  |
| Cajal et al (2023) [39] | PSG AHI >= 15 | CART | University Hospitals Leuven (Belgium) in-lab PSG | | | |  |
| Chen et al (2023) [20] | PSG AHI >= 5 | NN | MESA and MrOS | - | | SHHS 1 and 2 |  |
| Chen et al (2023) [20] | PSG AHI >= 15 | NN | MESA and MrOS | - | | SHHS 1 and 2 |  |
| Chen et al (2023) [20] | PSG AHI >= 30 | NN | MESA and MrOS | - | | SHHS 1 and 2 |  |
| Gutierrez-Tobal et al  (2021) [40] | HSAT AHI >= 5 | GB | SHHS1 | SHHS1 | | SHHS1 |  |
| Gutierrez-Tobal et al  (2021) [40] | HSAT AHI >= 5 | SVM | SHHS1 | SHHS1 | | SHHS1 |  |
| Gutierrez-Tobal et al  (2021) [40] | HSAT AHI >= 5 | CART | SHHS1 | SHHS1 | | SHHS1 |  |
| Gutierrez-Tobal et al  (2021) [40] | HSAT AHI >= 15 | GB | SHHS1 | SHHS1 | | SHHS1 |  |
| Gutierrez-Tobal et al  (2021) [40] | HSAT AHI >= 15 | SVM | SHHS1 | SHHS1 | | SHHS1 |  |
| Gutierrez-Tobal et al  (2021) [40] | HSAT AHI >= 15 | CART | SHHS1 | SHHS1 | | SHHS1 |  |
| Gutierrez-Tobal et al  (2021) [40] | HSAT AHI >= 30 | GB | SHHS1 | SHHS1 | | SHHS1 |  |
| Gutierrez-Tobal et al  (2021) [40] | HSAT AHI >= 30 | SVM | SHHS1 | SHHS1 | | SHHS1 |  |
| Gutierrez-Tobal et al  (2021) [40] | HSAT AHI >= 30 | CART | SHHS1 | SHHS1 | | SHHS1 |  |
| Gutierrez-Tobal et al  (2021) [40] | HSAT AHI >= 5 | GB | SHHS1 | SHHS1 | | RHUH at home PSG, 322 samples |  |
| Gutierrez-Tobal et al  (2021) [40] | HSAT AHI >= 5 | SVM | SHHS1 | SHHS1 | | RHUH at home PSG, 322 samples |  |
| Gutierrez-Tobal et al  (2021) [40] | HSAT AHI >= 5 | CART | SHHS1 | SHHS1 | | RHUH at home PSG, 322 samples |  |
| Gutierrez-Tobal et al  (2021) [40] | HSAT AHI >= 15 | GB | SHHS1 | SHHS1 | | RHUH at home PSG, 322 samples |  |
| Gutierrez-Tobal et al  (2021) [40] | HSAT AHI >= 15 | SVM | SHHS1 | SHHS1 | | RHUH at home PSG, 322 samples |  |
| Gutierrez-Tobal et al  (2021) [40] | HSAT AHI >= 15 | CART | SHHS1 | SHHS1 | | RHUH at home PSG, 322 samples |  |
| Gutierrez-Tobal et al  (2021) [40] | HSAT AHI >= 30 | GB | SHHS1 | SHHS1 | | RHUH at home PSG, 322 samples |  |
| Gutierrez-Tobal et al  (2021) [40] | HSAT AHI >= 30 | SVM | SHHS1 | SHHS1 | | RHUH at home PSG, 322 samples |  |
| Gutierrez-Tobal et al  (2021) [40] | HSAT AHI >= 30 | CART | SHHS1 | SHHS1 | | RHUH at home PSG, 322 samples |  |
| Kaimakamis et al  (2009) [41] | PSG AHI >= 5 | CART | G Papanikolaou Hospital | | | |  |
| Kaimakamis et al  (2009) [41] | PSG AHI >= 15 | CART | G Papanikolaou Hospital | | | |  |
| Kaimakamis et al  (2009) [41] | PSG AHI >= 30 | CART | G Papanikolaou Hospital | | | |  |
| Kuo et al (2025) [58] | PSG AHI >= 15 | GB | TVGH | | | | |
| Leong et al (2023) [42] | PSG AHI >= 5 | LR | Singapore Sleep Medicine Database | | | |  |
| Leong et al (2023) [42] | PSG AHI >= 5 | RF | Singapore Sleep Medicine Database | | | |  |
| Leong et al (2023) [42] | PSG AHI >= 5 | SVM | Singapore Sleep Medicine Database | | | |  |
| Leong et al (2023) [42] | PSG AHI >= 5 | GB | Singapore Sleep Medicine Database | | | |  |
| Leong et al (2023) [42] | PSG AHI >= 5 | NN | Singapore Sleep Medicine Database | | | |  |
| Leong et al (2023) [42] | PSG AHI >= 15 | LR | Singapore Sleep Medicine Database | | | |  |
| Leong et al (2023) [42] | PSG AHI >= 15 | RF | Singapore Sleep Medicine Database | | | |  |
| Leong et al (2023) [42] | PSG AHI >= 15 | SVM | Singapore Sleep Medicine Database | | | |  |
| Leong et al (2023) [42] | PSG AHI >= 15 | GB | Singapore Sleep Medicine Database | | | |  |
| Leong et al (2023) [42] | PSG AHI >= 15 | NN | Singapore Sleep Medicine Database | | | |  |
| Li et al (2021) [43] | PSG AHI >= 5 | LDA | Beijing Tongren Hospital | | | |  |
| Li et al (2021) [43] | PSG AHI >= 5 | SVM | Beijing Tongren Hospital | | | |  |
| Li et al (2021) [43] | PSG AHI >= 5 | CART | Beijing Tongren Hospital | | | |  |
| Li et al (2021) [43] | PSG AHI >= 5 | LR | Beijing Tongren Hospital | | | |  |
| Li et al (2021) [43] | PSG AHI >= 5 | NN | Beijing Tongren Hospital | | | |  |
| Li et al (2025) [57] | PSG AHI >= 5 | NN | SHHS1 | SHHS1 | | SHHS1 |  |
| Li et al (2025) [57] | PSG AHI >= 15 | NN | SHHS1 | SHHS1 | | SHHS1 |  |
| Li et al (2025) [57] | PSG AHI >= 30 | NN | SHHS1 | SHHS1 | | SHHS1 |  |
| Li et al (2025) [57] | PSG AHI >= 5 | NN | SHHS1 | SHHS1 | | SHHS2 |  |
| Li et al (2025) [57] | PSG AHI >= 15 | NN | SHHS1 | SHHS1 | | SHHS2 |  |
| Li et al (2025) [57] | PSG AHI >= 30 | NN | SHHS1 | SHHS1 | | SHHS2 |  |
| Ma et al (2019) [44] | PSG AHI >= 5 | SVM | UCD | | | |  |
| Ma et al (2019) [44] | PSG AHI >= 5 | GB | UCD | | | |  |
| Marcos et al (2007) [45] | PSG AHI>= 10 | NN | Clínico de Santiago de Compostela, Spain | | | |  |
| Marcos et al (2007) [45] | PSG AHI>= 10 | SVM | Clínico de Santiago de Compostela, Spain | | | |  |
| Marcos et al (2008) [46] | PSG AHI>= 10 | SVM | Clínico de Santiago de Compostela, Spain | | | |  |
| Marcos et al (2008) [47] | PSG AHI>= 10 | NN | Clínico de Santiago de Compostela, Spain | | | |  |
| Marcos et al (2009) [48] | PSG AHI>= 10 | SVM | Clínico de Santiago de Compostela, Spain | | | |  |
| Marcos et al (2010) [49] | PSG AHI>= 10 | NN | Clínico de Santiago de Compostela, Spain | | | |  |
| Muthukumaran et al (2025) [56] | PSG AHI >= 5 | NN | SHHS1 | SHHS1 | | SHHS1 |  |
| Muthukumaran et al (2025) [56] | PSG AHI >= 15 | NN | SHHS1 | SHHS1 | | SHHS1 |  |
| Muthukumaran et al (2025) [56] | PSG AHI >= 30 | NN | SHHS1 | SHHS1 | | SHHS1 |  |
| Muthukumaran et al (2025) [56] | PSG AHI >= 5 | NN | SHHS1 | SHHS1 | | SHHS2 |  |
| Muthukumaran et al (2025) [56] | PSG AHI >= 15 | NN | SHHS1 | SHHS1 | | SHHS2 |  |
| Muthukumaran et al (2025) [56] | PSG AHI >= 30 | NN | SHHS1 | SHHS1 | | SHHS2 |  |
| Muthukumaran et al (2025) [56] | PSG AHI >= 5 | NN | SHHS1 | SHHS1 | | CFS |  |
| Muthukumaran et al (2025) [56] | PSG AHI >= 15 | NN | SHHS1 | SHHS1 | | CFS |  |
| Muthukumaran et al (2025) [56] | PSG AHI >= 30 | NN | SHHS1 | SHHS1 | | CFS |  |
| Nikkonen et al (2019) [50] | HSAT AHI >= 5 | NN | Unisalkku Data (Kuopio University Hospital, Finland) | | | |  |
| Nikkonen et al (2019) [50] | HSAT AHI >= 10 | NN | Unisalkku Data (Kuopio University Hospital, Finland) | | | |  |
| Nikkonen et al (2019) [50] | HSAT AHI >= 15 | NN | Unisalkku Data (Kuopio University Hospital, Finland) | | | |  |
| Nikkonen et al (2019) [50] | HSAT AHI >= 5 | NN | Unisalkku Data | Unisalkku Data | | Embletta Data (Kuopio University Hospital, Finland) |  |
| Nikkonen et al (2019) [50] | HSAT AHI >= 10 | NN | Unisalkku Data | Unisalkku Data | | Embletta Data (Kuopio University Hospital, Finland) |  |
| Nikkonen et al (2019) [50] | HSAT AHI >= 15 | NN | Unisalkku Data | Unisalkku Data | | Embletta Data (Kuopio University Hospital, Finland) |  |
| Peng et al (2024) [51] | PSG AHI >= 5 | NN | FAH and CMH | | | |  |
| Peng et al (2024) [51] | PSG AHI >= 10 | NN | FAH and CMH | | | |  |
| Peng et al (2024) [51] | PSG AHI >= 15 | NN | FAH and CMH | | | |  |
| Polat et al (2008) [52] | PSG AHI >= 5 | NN | Selcuk University, Konya, Turkey | | | |  |
| Polat et al (2008) [52] | PSG AHI >= 15 | NN | Selcuk University, Konya, Turkey | | | |  |
| Polat et al (2008) [52] | PSG AHI >= 30 | NN | Selcuk University, Konya, Turkey | | | |  |
| Wu et al (2018) [53] | PSG AHI >= 5 | SVM | CGMH, Taoyuan, Taiwan | | | |  |
| Wu et al (2018) [53] | PSG AHI >= 15 | SVM | CGMH, Taoyuan, Taiwan | | | |  |
| Wu et al (2018) [53] | PSG AHI >= 30 | SVM | CGMH, Taoyuan, Taiwan | | | |  |
| Wu et al (2025) [55] | PSG AHI >= 5 | NN | MESA and MrOS | - | | SHHS1 |  |
| Wu et al (2025) [55] | PSG AHI >= 15 | NN | MESA and MrOS | - | | SHHS1 |  |
| Wu et al (2025) [55] | PSG AHI >= 30 | NN | MESA and MrOS | - | | SHHS1 |  |
| Wu et al (2025) [55] | PSG AHI >= 5 | NN | MESA and MrOS | - | | SHHS2 |  |
| Wu et al (2025) [55] | PSG AHI >= 15 | NN | MESA and MrOS | - | | SHHS2 |  |
| Wu et al (2025) [55] | PSG AHI >= 30 | NN | MESA and MrOS | - | | SHHS2 |  |
| Zhang et al (2023) [54] | PSG AHI >= 5 | SVM | UCD | | | |  |
| Zhang et al (2023) [54] | PSG AHI >= 5 | NN | UCD | | | |  |
| Zhang et al (2023) [54] | PSG AHI >= 5 | RF | UCD | | | |  |
| Zhang et al (2023) [54] | PSG AHI >= 5 | LDA | UCD | | | |  |

Abbreviations: PSG, polysomnography; HSAT, home sleep apnea test; AHI, apnea-hypopnea index; SVM, Support Vector Machine; NN, Neural Network; RF, Random Forest; LDA, Linear Discriminant Analysis; GB, Gradient Boosting; LR, Logistic Regression; CART, Classification and Regression Tree; RHUH, Río Hortega University Hospital of Valladolid (Spain); EPISONO, São Paulo Epidemiologic Sleep Study; MESA, Multi-

Ethnic Study of Atherosclerosis; MrOS, Osteoporotic Fractures in Men Study; SHHS1, Sleep Heart Health Study Visit 1; SHHS2, Sleep Heart Health Study Visit 2; TVGH, Taiwan Veterans General Hospital; UCD, University College of Dublin; CFS, Cleaveland Family Study; FAH, Sleep Center of the First Affiliated Hospital; CMH, Sun Yat-sen University and the Integrative Department of Guangdong Province Traditional Chinese Medical Hospital; CGMH, Chang Gung Memorial Hospital;

Supplemental Table S5**: Comparing the sensitivity and specificity of AI vs ODI**

|  | Sensitivity (%) | | Specificity (%) | | |
| --- | --- | --- | --- | --- | --- |
| AHI cut-off | AI[35] | ODI3[35] | | AI[35] | ODI3[35] |
| AHI ≥10 | 91 | 60 | | 79 | 91 |

Supplemental Table S6**: Comparing the specificity of AI vs HSAT at varying AHI cut-offs**

|  | Specificity (%) | | | Sensitivity (%) | |  |
| --- | --- | --- | --- | --- | --- | --- |
| AHI cut-off | AI | HSAT[51] | AI | | HSAT[51] |  |
| AHI ≥5 | 78 | 43 | 93 | | 94 |  |
| AHI ≥15 | 86 | 72 | 89 | | 92 |  |
| AHI ≥30 | 94 | 87 | 89 | | 74 |  |

References:

12. Andrés-Blanco AM, Álvarez D, Crespo A, et al. Assessment of automated analysis of portable oximetry as a screening test for moderate-to-severe sleep apnea in patients with chronic obstructive pulmonary disease. PLoS ONE. 2017;12(11):e0188094. [doi: 10.1371/journal.pone.0188094] [Medline: 29176802]

13. Behar JA, Palmius N, Li Q, et al. Feasibility of single channel oximetry for mass screening of obstructive sleep apnea. EClinicalMedicine. 2019;11:81-88. [doi: 10.1016/j.eclinm.2019.05.015] [Medline: 31317133]

20. Chen JW, Liu CM, Wang CY, et al. A deep neural network-based model for OSA severity classification using unsegmented peripheral oxygen saturation signals. Eng Appl Artif Intell. Jun 2023;122:106161. [doi: 10.1016/j.engappai.2023.106161]

37. Álvarez D, Cerezo-Hernández A, Crespo A, et al. A machine learning-based test for adult sleep apnoea screening at home using oximetry and airflow. Sci Rep. Mar 24, 2020;10(1):5332. [doi: 10.1038/s41598-020-62223-4] [Medline: 32210294]

38. Alvarez D, Gutierrez-Tobal GC, Vaquerizo-Villar F, et al. Automated analysis of unattended portable oximetry by means of bayesian neural networks to assist in the diagnosis of sleep apnea. Presented at: 2016 Global Medical Engineering Physics Exchanges/Pan American Health Care Exchanges (GMEPE/PAHCE); Apr 4, 2026 to Apr 9, 2016:79-82; Madrid, Spain. 2016.[doi: 10.1109/GMEPE-PAHCE.2016.7504628]

39. Cajal D, Gil E, Laguna P, et al. Obstructive sleep apnea screening by joint saturation signal analysis and PPG-derived pulse rate oscillations. IEEE J Biomed Health Inform. Nov 10, 2023;PP. [doi: 10.1109/JBHI.2023.3331947] [Medline: 37948138]

40. Gutiérrez-Tobal GC, Álvarez D, Vaquerizo-Villar F, et al. Ensemble-learning regression to estimate sleep apnea severity using at-home oximetry in adults. Appl Soft Comput. Nov 2021;111:107827. [doi: 10.1016/j.asoc.2021.107827] [Medline: 39544517]

41. Kaimakamis E, Bratsas C, Sichletidis L, Karvounis C, Maglaveras N. Screening of patients with obstructive sleep apnea syndrome using C4.5 algorithm based on non linear analysis of respiratory signals during sleep. Annu Int Conf IEEE Eng Med Biol Soc. 2009;2009:3465-3469. [doi: 10.1109/IEMBS.2009.5334605] [Medline: 19964987]

42. Leong ZH, Loh SRH, Leow LC, Ong TH, Toh ST. A machine learning approach for the diagnosis of obstructive sleep apnoea using oximetry, demographic and anthropometric data. Singapore Med J. Apr 1, 2025;66(4):195-201. [doi: 10.4103/singaporemedj.SMJ-2022-170] [Medline: 37171440]

43. Li Z, Li Y, Zhao G, Zhang X, Xu W, Han D. A model for obstructive sleep apnea detection using a multi-layer feed-forward neural network based on electrocardiogram, pulse oxygen saturation, and body mass index. Sleep Breath. Dec 2021;25(4):2065-2072. [doi: 10.1007/s11325-021-02302-6] [Medline: 33754247]

44. Ma B, Wu Z, Li S, et al. A svm-based algorithm to diagnose sleep apnea. Presented at: 2019 IEEE International Conference on Bioinformatics and Biomedicine (BIBM). Nov 18-21, 2019:Piscataway, NJ. 1556-1560; San Diego, CA, USA. Nov 2019.[doi: 10.1109/BIBM47256.2019.8983201]

45. Marcos JV, Hornero R, Alvarez D, Del Campo F, López M. Applying neural network classifiers in the diagnosis of the obstructive sleep apnea syndrome from nocturnal pulse oximetric recordings. Annu Int Conf IEEE Eng Med Biol Soc. 2007;2007:5174-5177. [doi: 10.1109/IEMBS.2007.4353507] [Medline: 18003173]

46. Marcos JV, Hornero R, Alvarez D, del Campo F, López M, Zamarrón C. Radial basis function classifiers to help in the diagnosis of the obstructive sleep apnoea syndrome from nocturnal oximetry. Med Biol Eng Comput. Apr 2008;46(4):323-332. [doi: 10.1007/s11517-007-0280-0] [Medline: 17968604]

47. Marcos JV, Hornero R, Alvarez D, Del Campo F, Zamarrón C, López M. Utility of multilayer perceptron neural network classifiers in the diagnosis of the obstructive sleep apnoea syndrome from nocturnal oximetry. Comput Methods Programs Biomed. Oct 2008;92(1):79-89. [doi: 10.1016/j.cmpb.2008.05.006] [Medline: 18672313]

48. Marcos JV, Hornero R, Alvarez D, Del Campo F, Zamarron C. Automated prediction of the apnea-hypopnea index from nocturnal oximetry recordings. Presented at: 2009 Annual International Conference of the IEEE Engineering in Medicine and Biology Society (EMBC 2009). Sep 2-6, 2009:IEEE. 5547-5550; Minneapolis, MN. 2009.[doi: 10.1109/IEMBS.2009.5333731]

49. Marcos JV, Hornero R, Alvarez D, Nabney IT, Del Campo F, Zamarrón C. The classification of oximetry signals using Bayesian neural networks to assist in the detection of obstructive sleep apnoea syndrome. Physiol Meas. Mar 2010;31(3):375-394. [doi: 10.1088/0967-3334/31/3/007] [Medline: 20130342]

50. Nikkonen S, Afara IO, Leppänen T, Töyräs J. Artificial neural network analysis of the oxygen saturation signal enables accurate diagnostics of sleep apnea. Sci Rep. Sep 13, 2019;9(1):13200. [doi: 10.1038/s41598-019-49330-7] [Medline: 31519927]

51. Peng D, Yue H, Tan W, et al. A bimodal feature fusion convolutional neural network for detecting obstructive sleep apnea/hypopnea from nasal airflow and oximetry signals. Artif Intell Med. Apr 2024;150:102808. [doi: 10.1016/j.artmed.2024.102808] [Medline: 38553148]

52. Polat K, Yosunkaya S, Güneş S. Pairwise ANFIS approach to determining the disorder degree of obstructive sleep apnea syndrome. J Med Syst. Oct 2008;32(5):379-387. [doi: 10.1007/s10916-008-9143-y] [Medline: 18814494]

53. Wu HT, Wu JC, Huang PC, et al. Phenotype-based and self-learning inter-individual sleep apnea screening with a level IV-like monitoring system. Front Physiol. 2018;9:723. [doi: 10.3389/fphys.2018.00723] [Medline: 30013479]

54. Zhang M, Dong C, Zhang D, Tseng ML, Wei J. An intelligent classification diagnosis based on blood oxygen saturation signals for medical data security including COVID-19 in industry 5.0. IEEE Trans Ind Inf. Mar 2023;19(3):3310-3320. [doi: 10.1109/TII.2022.3152809]

55. Wu YC, Yeh CY, Lin CC. Severity prediction of obstructive sleep apnea using transformed 2D oxygen saturation signals. Sens Mater. 2025;37(12):5535. [doi: 10.18494/SAM5765]

56. Muthukumaran MP, Nnamdi MC, Tamo JB, Purnell C, Wang MD. Developing an attention-based deep learning framework for obstructive sleep apnea detection using single-channel oximetry signal. Presented at: 2025 IEEE EMBS International Conference on Biomedical and Health Informatics (BHI). Oct 26-29, 2025:IEEE. 1-7; Atlanta, GA, USA. 2025.[doi: 10.1109/BHI67747.2025.11269547]

57. Li C, He S, Xu X, Wang Z. Deep model based on Mamba fusion multi-scale convolution LSTM for OSA severity grading. Appl Sci (Basel). Dec 10, 2025;15(24):12990. [doi: 10.3390/app152412990]

58. Kuo NY, Tsai HJ, Tsai SJ, Yang AC. Efficient screening in obstructive sleep apnea using sequential machine learning models, questionnaires, and pulse oximetry signals: mixed methods study. J Med Internet Res. Dec 19, 2024;26:e51615. [doi: 10.2196/51615] [Medline: 39699950]
