# Supplementary material for: Artificial Intelligence Diagnosis of Obstructive Sleep Apnea Using Overnight Pulse Oximetry: A Systematic Review and Bayesian Meta-Analysis
Source: J Med Internet Res. 2026 Jul 8;28:e80349. doi: 10.2196/80349 (PMC13344538; doi:10.2196/80349)
Supplement: Checklist 2 [file jmir-v28-e80349-s003.docx]

**PRISMA-S Checklist**

| **Section/topic** | **#** | **Checklist item** | **Location(s) Reported** |
| --- | --- | --- | --- |
| **INFORMATION SOURCES AND METHODS** | | | |
| Database name | 1 | Name each individual database searched, stating the platform for each. | Page 6, Line 171-179  (under *Methods*: *Information Sources* section) |
| Multi-database searching | 2 | If databases were searched simultaneously on a single platform, state the name of the platform, listing all of the databases searched. | NA (Each database was searched independently and separately as detailed in Methods: Information sources section, Line 178) |
| Study registries | 3 | List any study registries searched. | NA (No study registry was searched, as detailed in Methods: Search strategy, Line 196-197) |
| Online resources and browsing | 4 | Describe any online or print source purposefully searched or browsed (e.g., tables of contents, print conference proceedings, web sites), and how this was done. | NA (No online browsing was performed, as detailed in Methods: Search strategy, Line 196-197) |
| Citation searching | 5 | Indicate whether cited references or citing references were examined, and describe any methods used for locating cited/citing references (e.g., browsing reference lists, using a citation index, setting up email alerts for references citing included studies). | NA (No citation searching was performed, as detailed in Methods: Search strategy, Line 196-197) |
| Contacts | 6 | Indicate whether additional studies or data were sought by contacting authors, experts, manufacturers, or others. | NA (No author contact was used for searching, as detailed in Methods: Search strategy, Line 196-197) |
| Other methods | 7 | Describe any additional information sources or search methods used. | NA (No other search methods were used, as detailed in Methods: Search strategy, Line 196-197) |
| **SEARCH STRATEGIES** | | | |
| Full search strategies | 8 | Include the search strategies for each database and information source, copied and pasted exactly as run. | Page 6, Line 181 – 197 (under *Methods*: *Search Strategy* section) and  Supplementary Material (under Supplemental Methods: Search Strategy) |
| Limits and restrictions | 9 | Specify that no limits were used, or describe any limits or restrictions applied to a search (e.g., date or time period, language, study design) and provide justification for their use. | Page 6, Line 193-197  (under *Methods*: *Search Strategy* section) and  Supplementary Material (under Supplemental Methods: Search Strategy) |
| Search filters | 10 | Indicate whether published search filters were used (as originally designed or modified), and if so, cite the filter(s) used. | NA (No search filters were used as detailed in Methods: Search strategy section line 196-197) |
| Prior work | 11 | Indicate when search strategies from other literature reviews were adapted or reused for a substantive part or all of the search, citing the previous review(s). | Page 6, Line 181-184 (under *Methods*: *Search Strategy* section) |
| Updates | 12 | Report the methods used to update the search(es) (e.g., rerunning searches, email alerts). | NA (Search was performed till 3 Jan 2026 as detailed in Methods: Information sources line 177-178 which is within 6 months from publication date) |
| Dates of searches | 13 | For each search strategy, provide the date when the last search occurred. | Page 6, Line 177-178 (under *Methods*: *Information Sources* section) |
| **PEER REVIEW** | | | |
| Peer review | 14 | Describe any search peer review process. | NA (None were used, as detailed in Methods: Search strategy line 197) |
| **MANAGING RECORDS** | | | |
| Total Records | 15 | Document the total number of records identified from each database and other information sources. | Page 6, Line 199-204 (under *Methods*: *Managing Records* section) |
| Deduplication | 16 | Describe the processes and any software used to deduplicate records from multiple database searches and other information sources. | Page 6, Line 199-204 (under *Methods*: *Managing Records* section) |
|  |  |  |  |
| PRISMA-S: An Extension to the PRISMA Statement for Reporting Literature Searches in Systematic Reviews | | |  |
| Rethlefsen ML, Kirtley S, Waffenschmidt S, Ayala AP, Moher D, Page MJ, Koffel JB, PRISMA-S Group. | | |  |
| Last updated February 27, 2020. | |  |  |
